# Supplementary figures and images for: The molecular coupling between substrate recognition and ATP turnover in a AAA+ hexameric helicase loader
Source: eLife. 2021 May 26;10:e64232. doi: 10.7554/eLife.64232 (PMC8213410; doi:10.7554/eLife.64232)

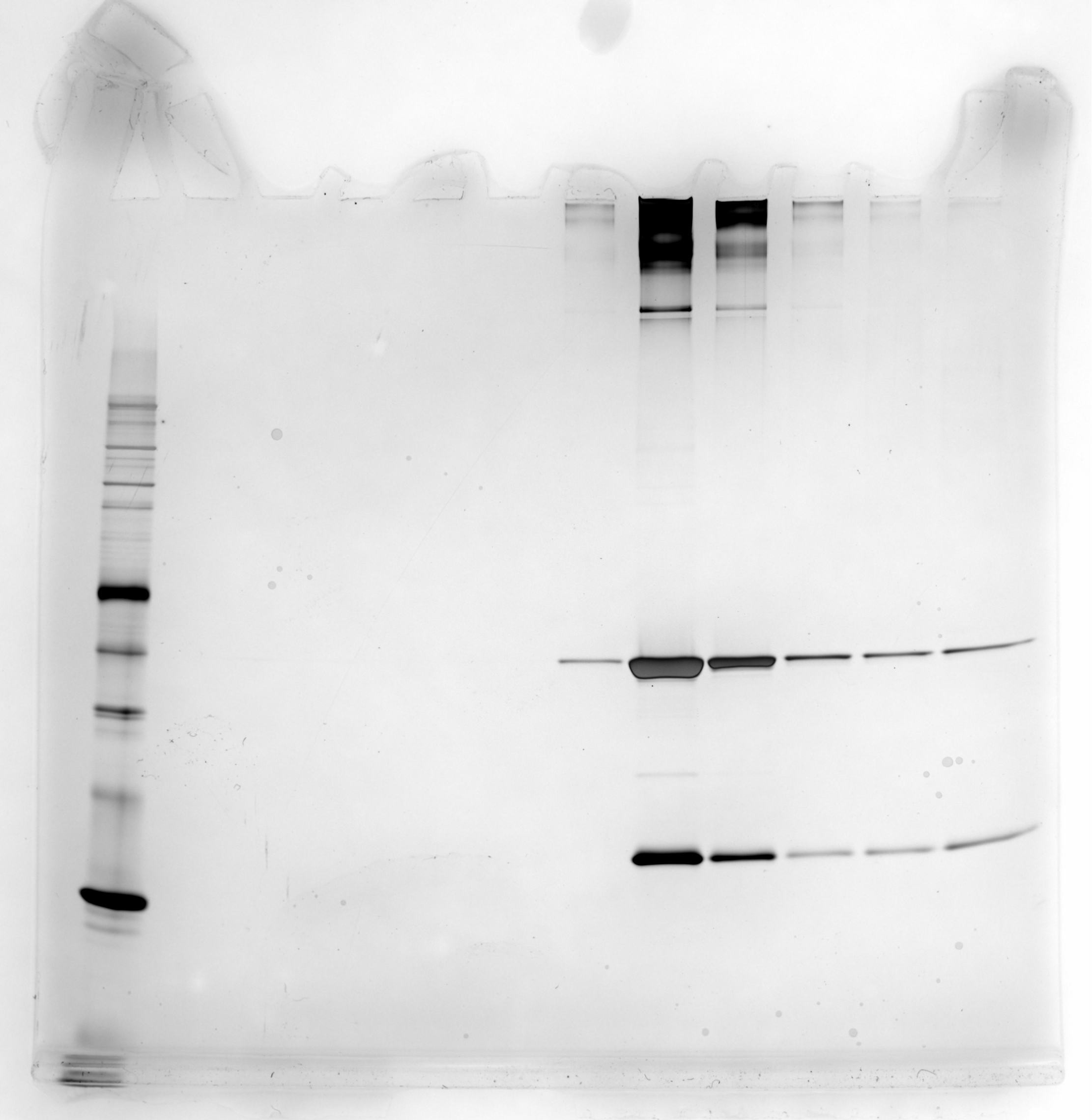

Supplement: Figure 2—source data 1. [file elife-64232-fig2-data1.zip › Fig 2/Fig 2C/051717 WT1.tif]

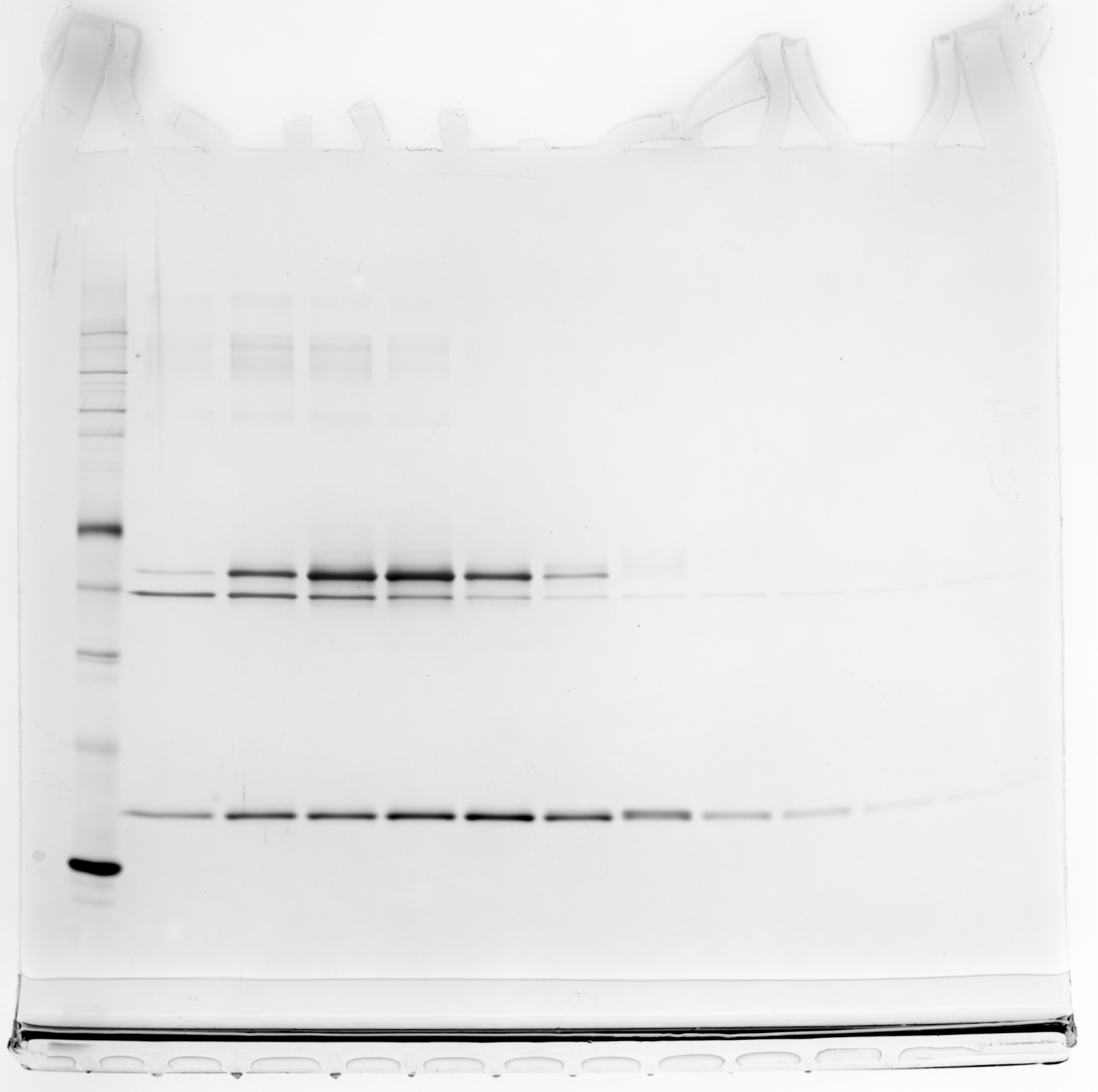

Supplement: Figure 2—source data 1. [file elife-64232-fig2-data1.zip › Fig 2/Fig 2C/051717 WT gel2.tif]

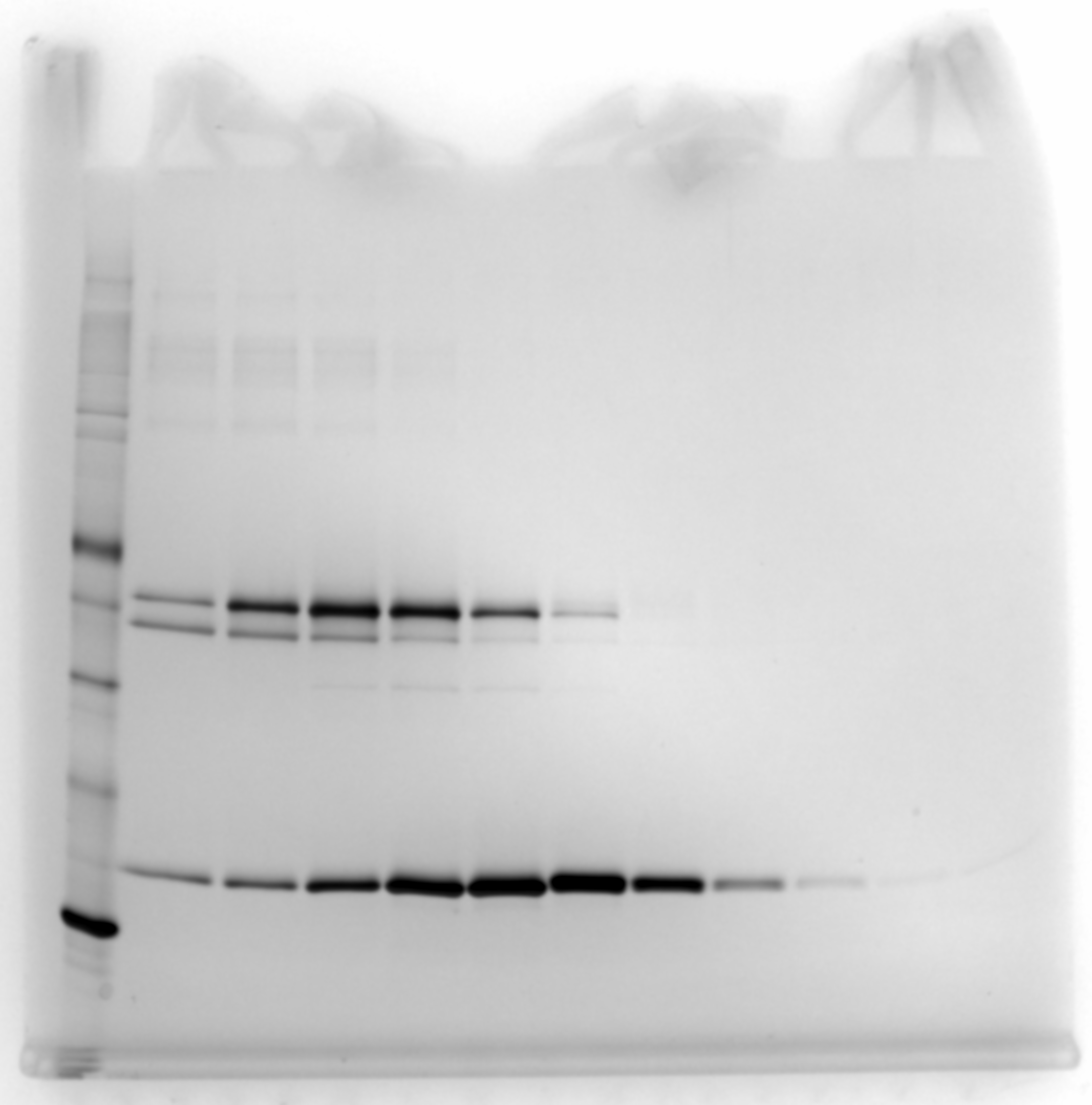

Supplement: Figure 2—figure supplement 1—source data 1. [file elife-64232-fig2-figsupp1-data1.zip › Fig2 S1/Fig2 S1F/100417 R237D gel 2.tif]

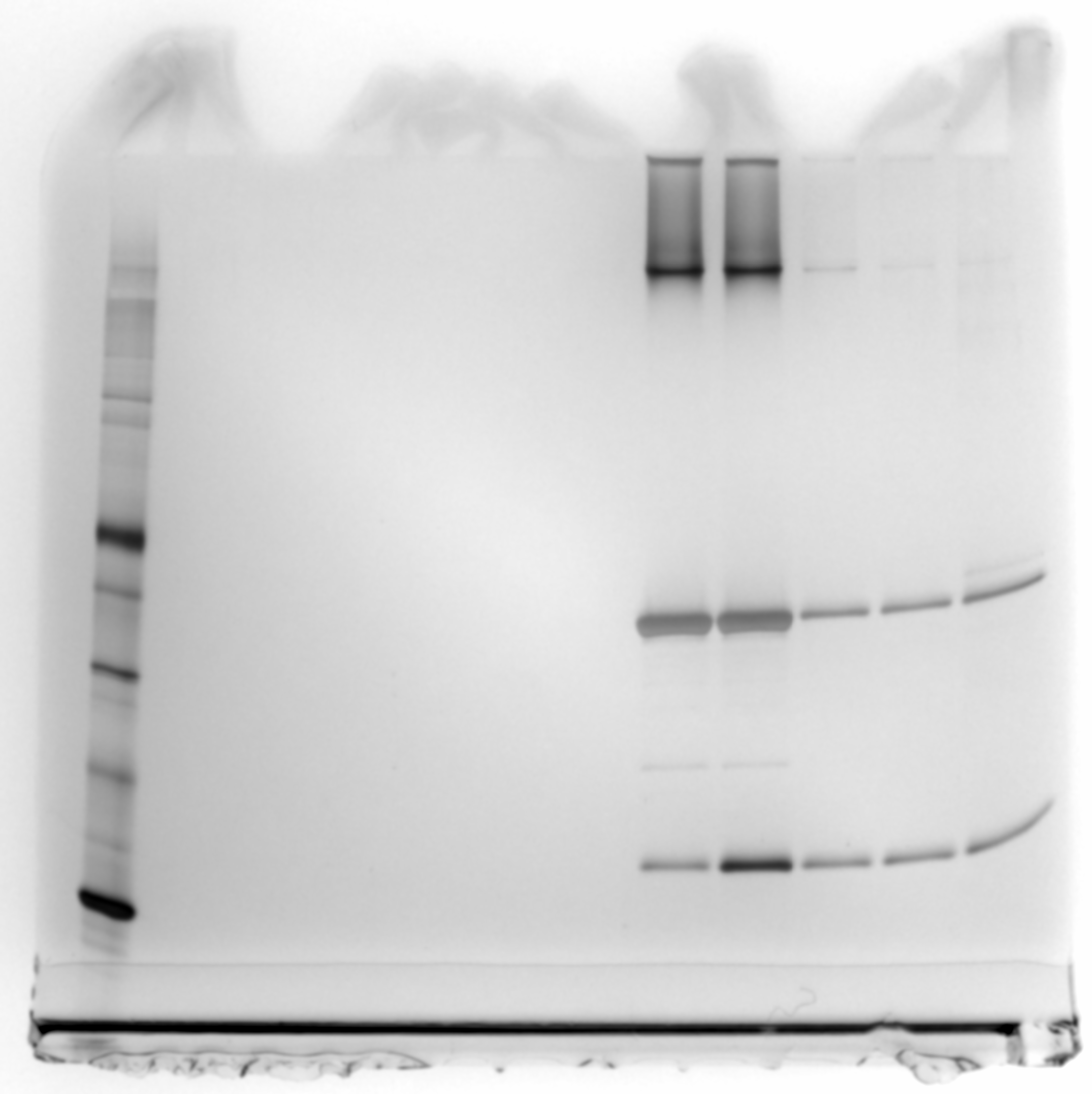

Supplement: Figure 2—figure supplement 1—source data 1. [file elife-64232-fig2-figsupp1-data1.zip › Fig2 S1/Fig2 S1F/100417 R237D gel 1.tif]

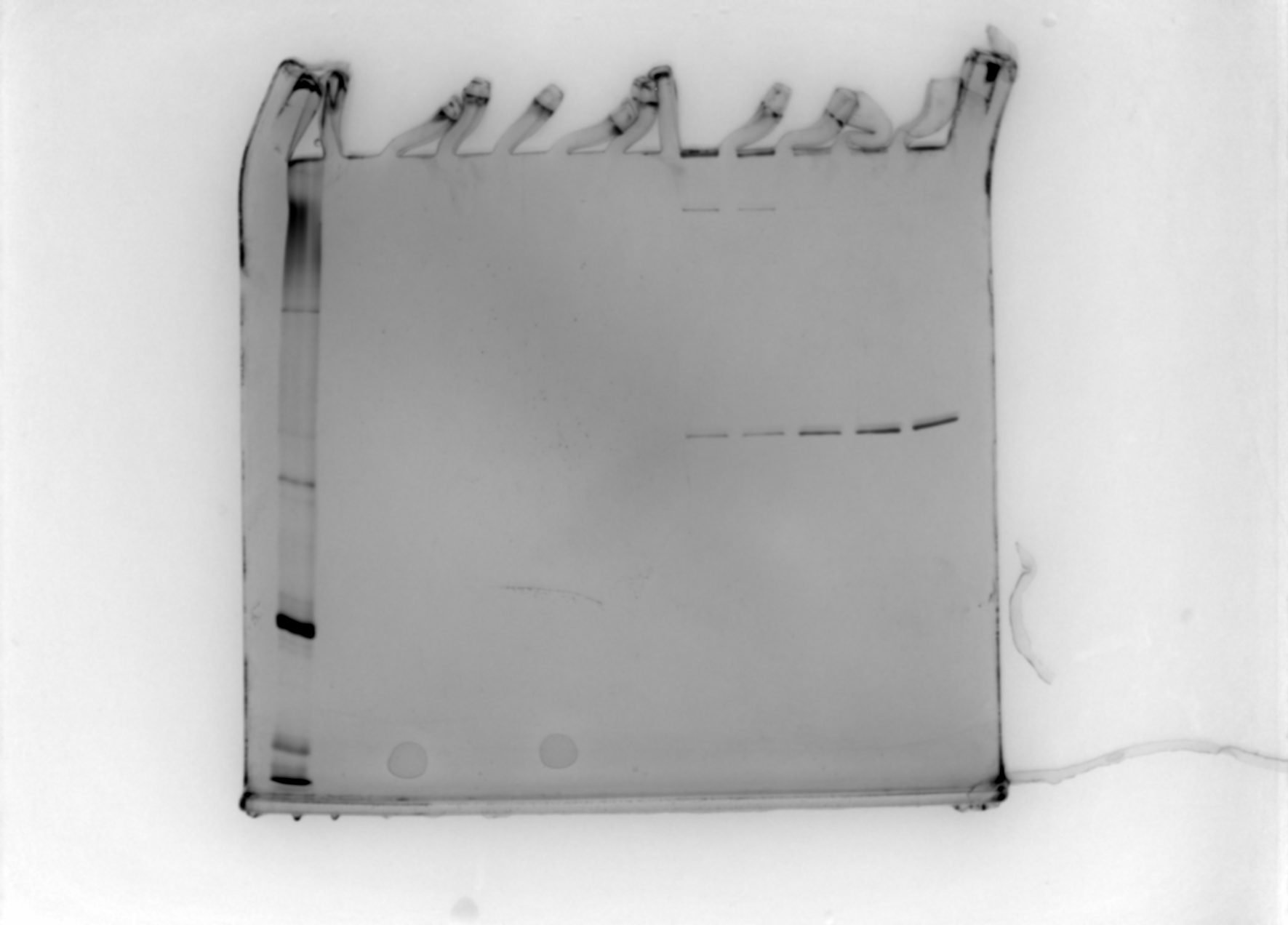

Supplement: Figure 2—figure supplement 1—source data 1. [file elife-64232-fig2-figsupp1-data1.zip › Fig2 S1/Fig2 S1A/20200919_B1.png]

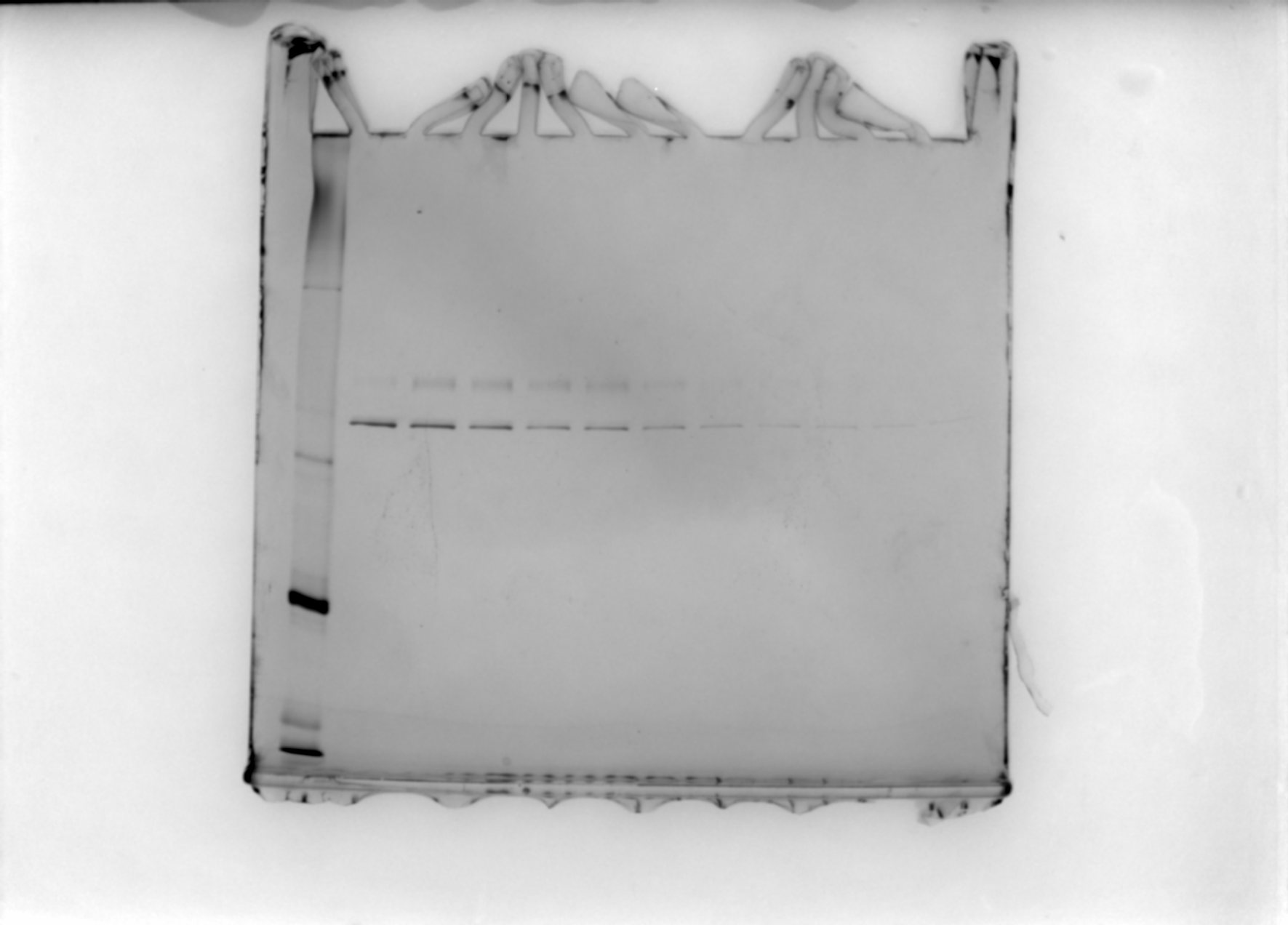

Supplement: Figure 2—figure supplement 1—source data 1. [file elife-64232-fig2-figsupp1-data1.zip › Fig2 S1/Fig2 S1A/20200919_B2.png]

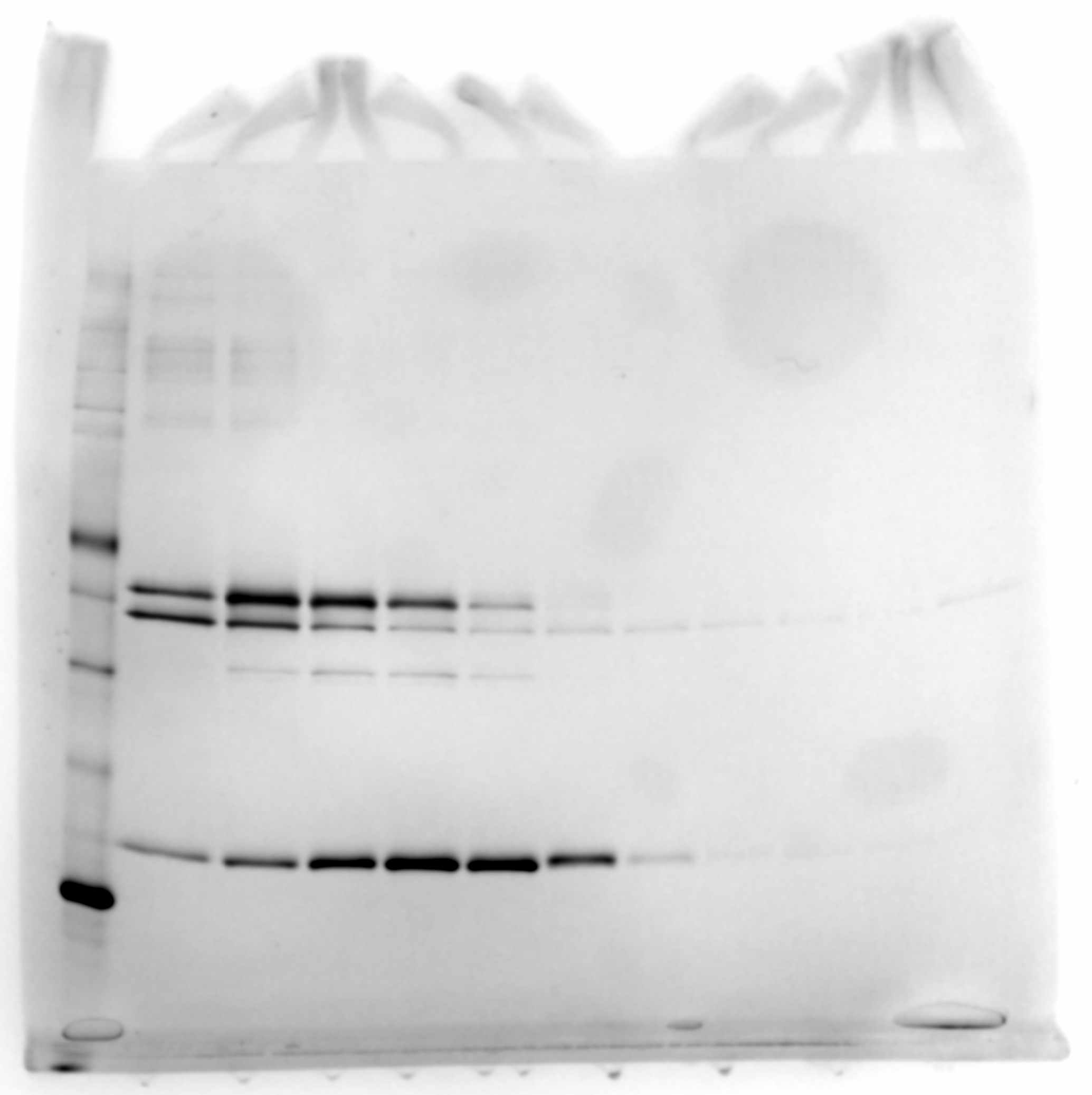

Supplement: Figure 2—figure supplement 1—source data 1. [file elife-64232-fig2-figsupp1-data1.zip › Fig2 S1/Fig2 S1G/101717 K112R gel 2.tif]

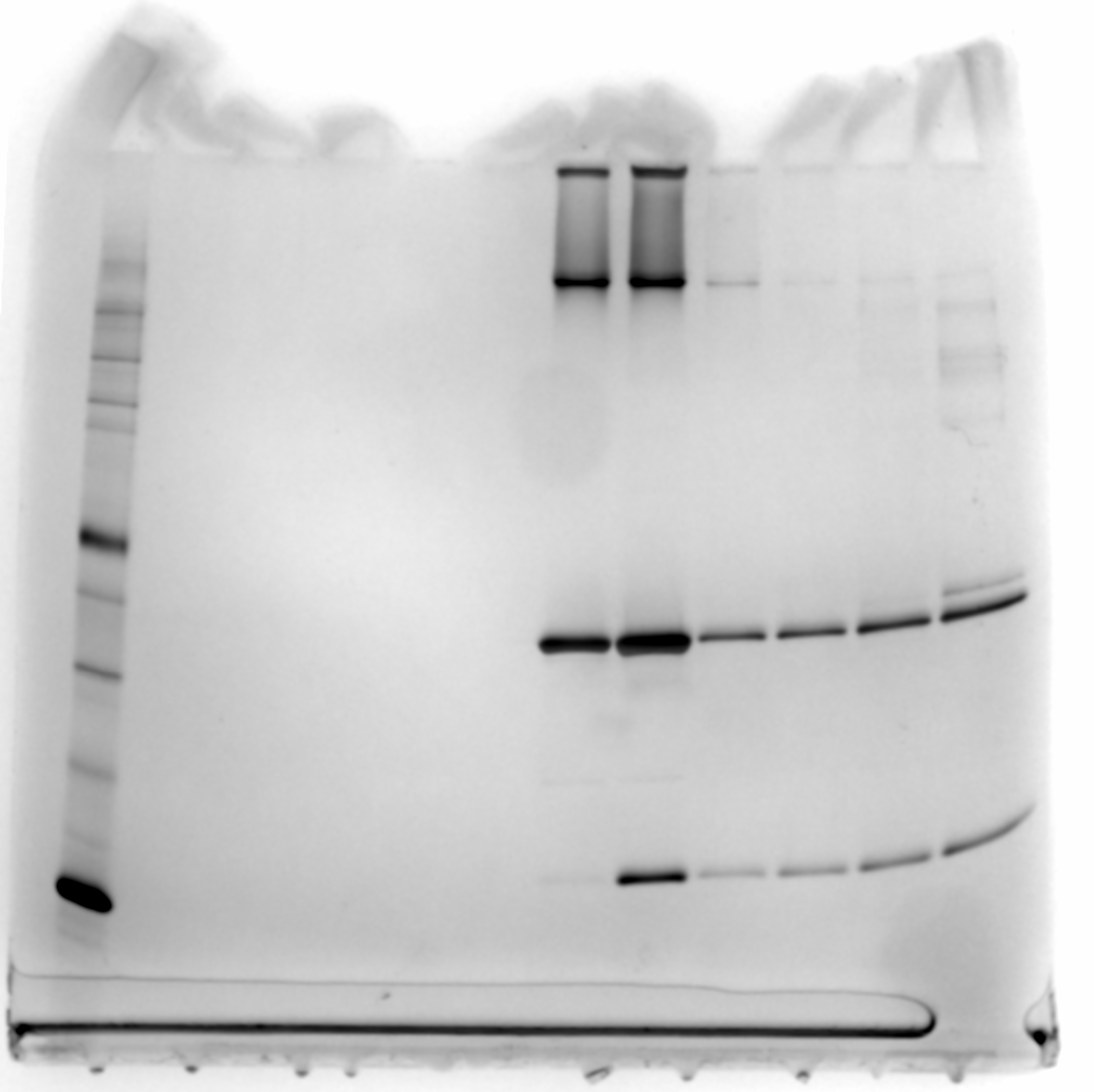

Supplement: Figure 2—figure supplement 1—source data 1. [file elife-64232-fig2-figsupp1-data1.zip › Fig2 S1/Fig2 S1G/101717 K112R gel 1.tif]

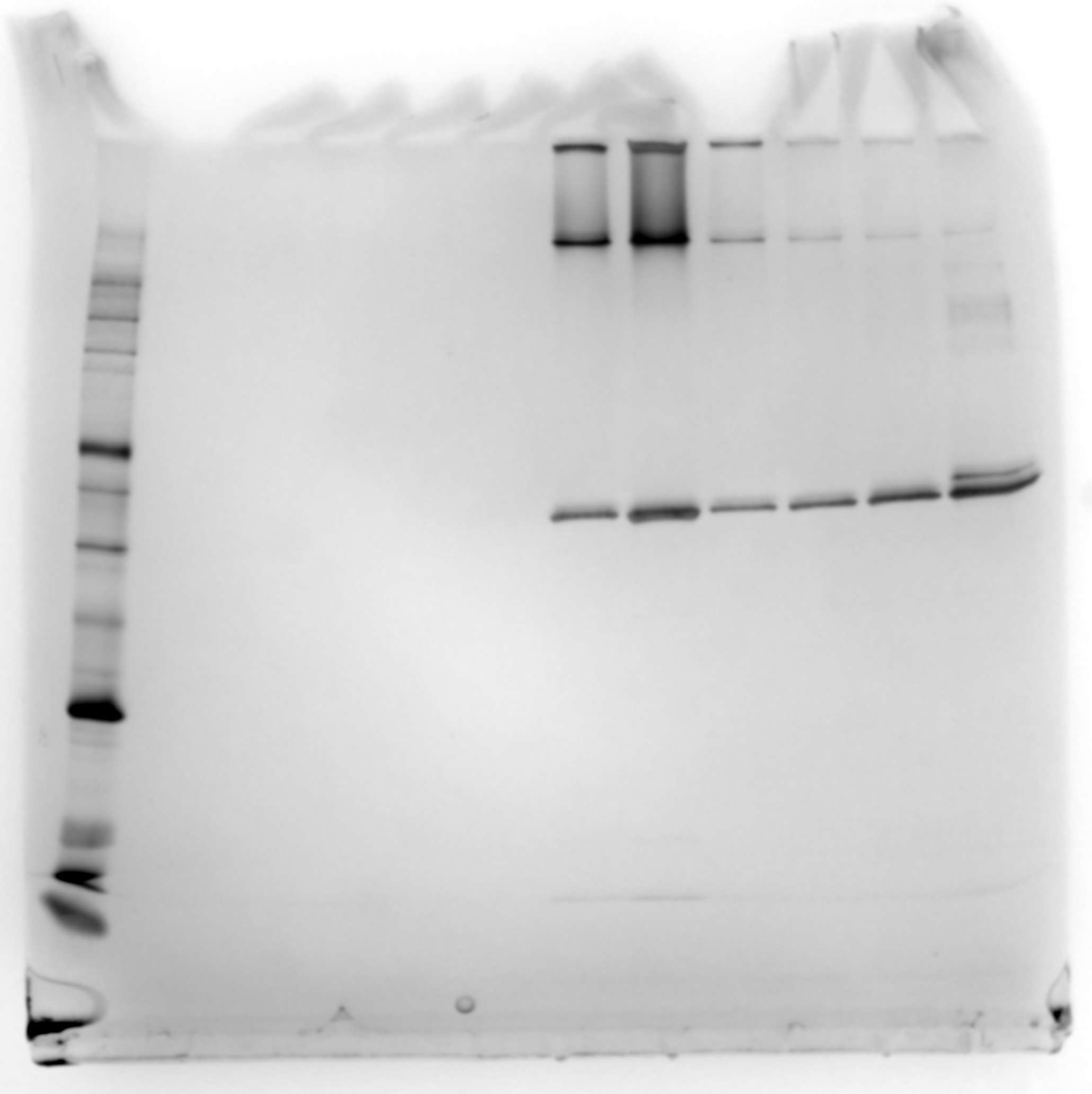

Supplement: Figure 2—figure supplement 1—source data 1. [file elife-64232-fig2-figsupp1-data1.zip › Fig2 S1/Fig2 S1B/101717 NTD gel 1.tif]

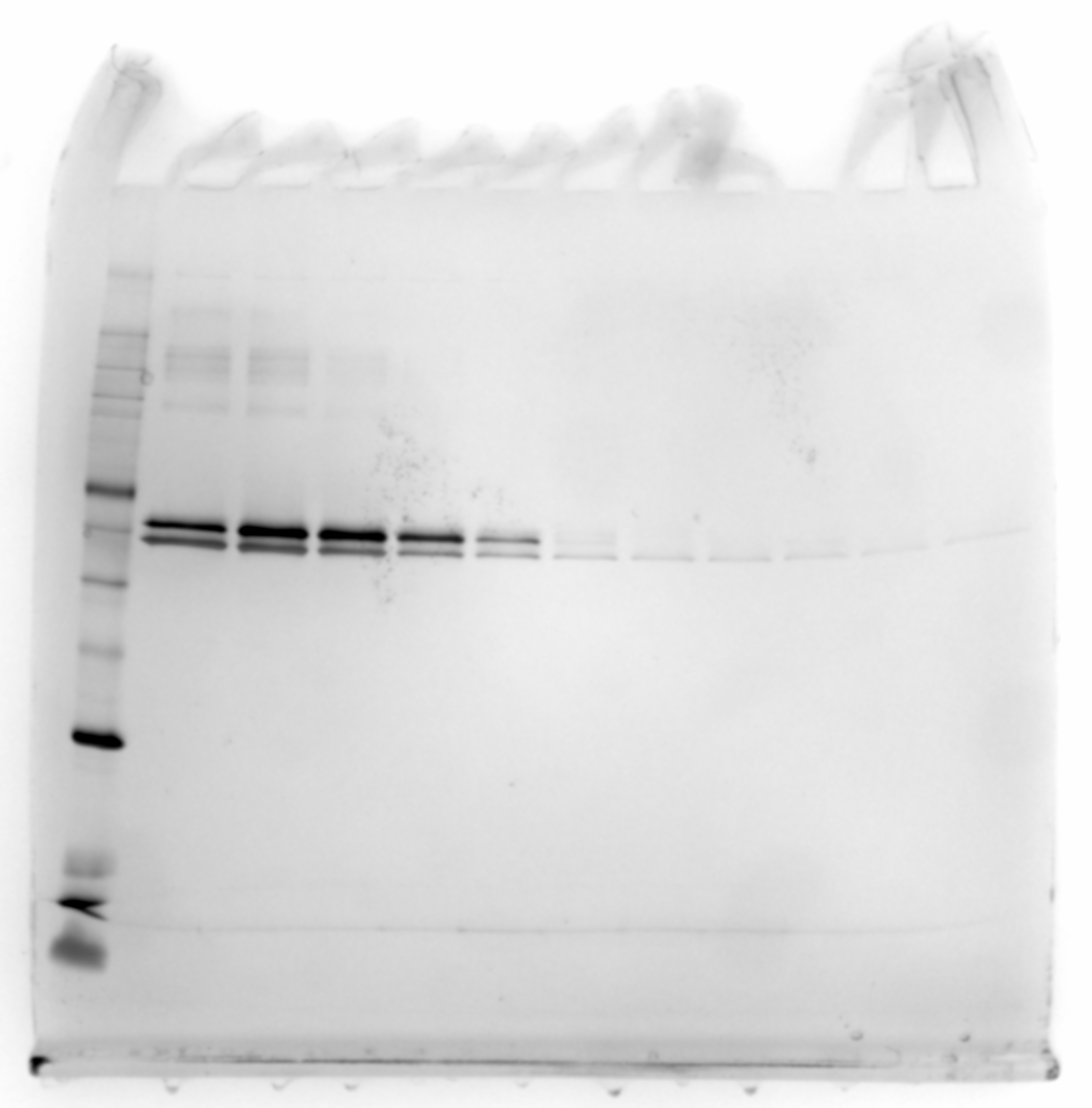

Supplement: Figure 2—figure supplement 1—source data 1. [file elife-64232-fig2-figsupp1-data1.zip › Fig2 S1/Fig2 S1B/101717 NTD gel 2.tif]

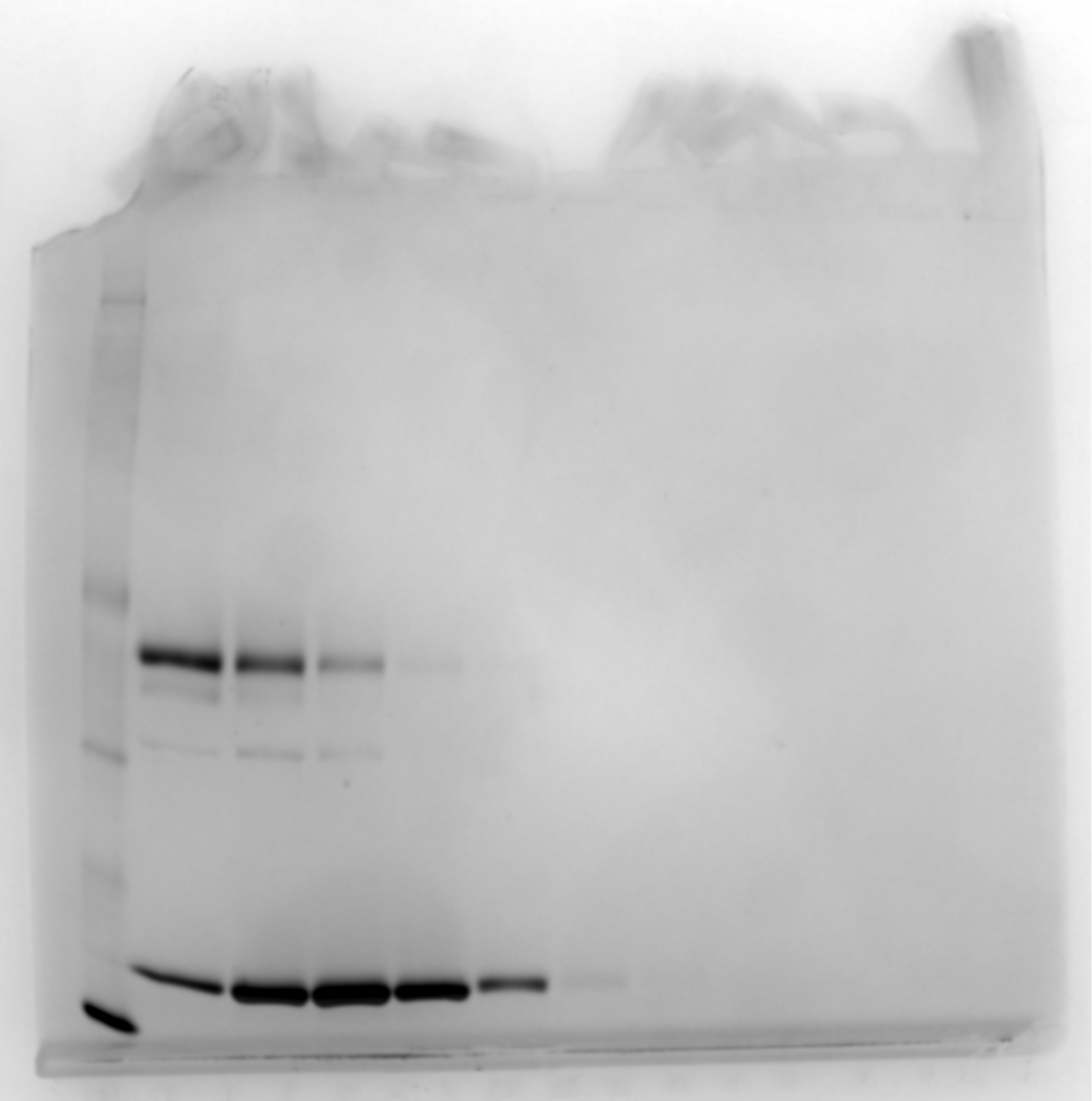

Supplement: Figure 2—figure supplement 1—source data 1. [file elife-64232-fig2-figsupp1-data1.zip › Fig2 S1/Fig2 S1E/110717 R220D gel 2.tif]

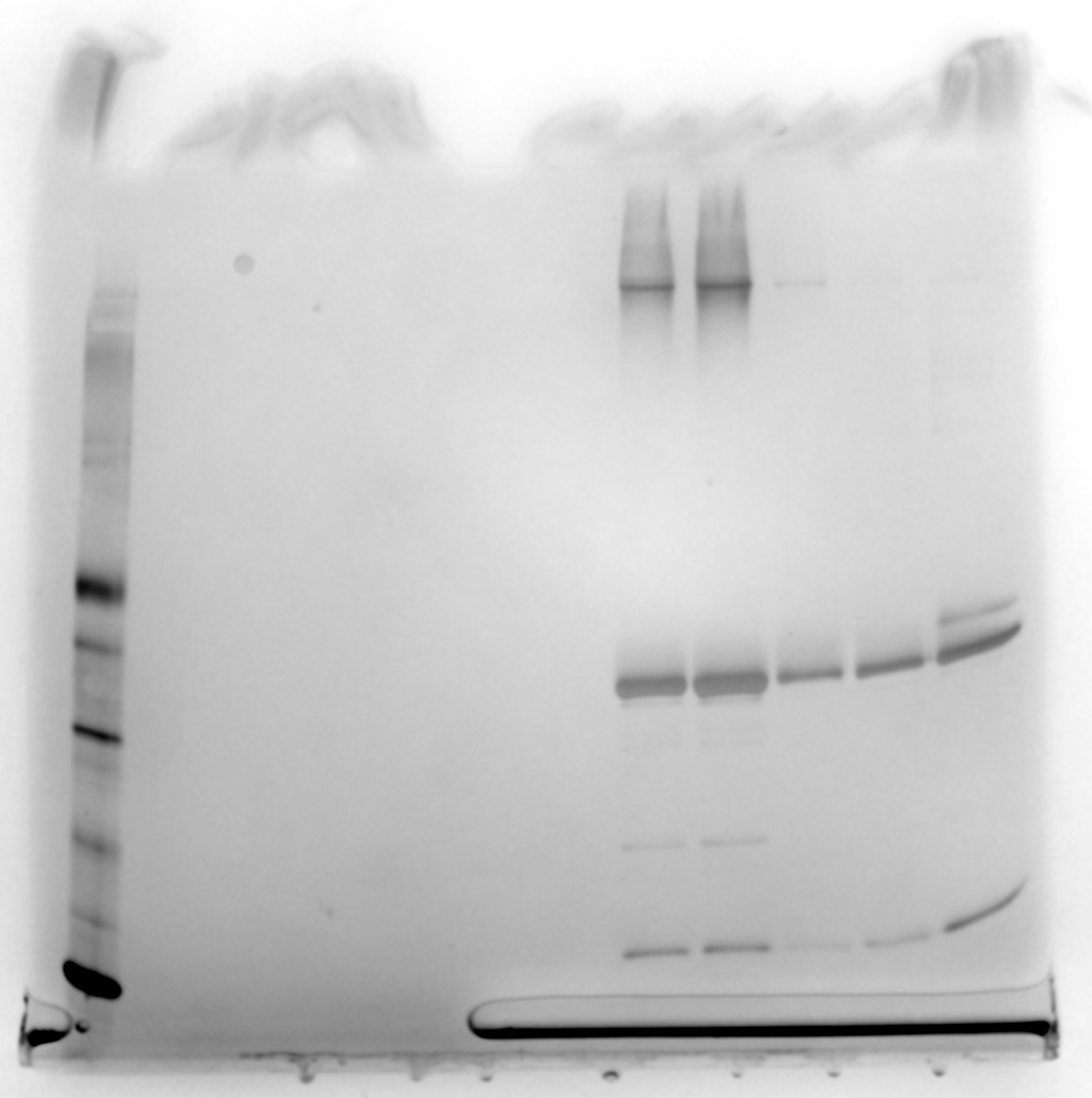

Supplement: Figure 2—figure supplement 1—source data 1. [file elife-64232-fig2-figsupp1-data1.zip › Fig2 S1/Fig2 S1E/110217 R220D gel 1.tif]

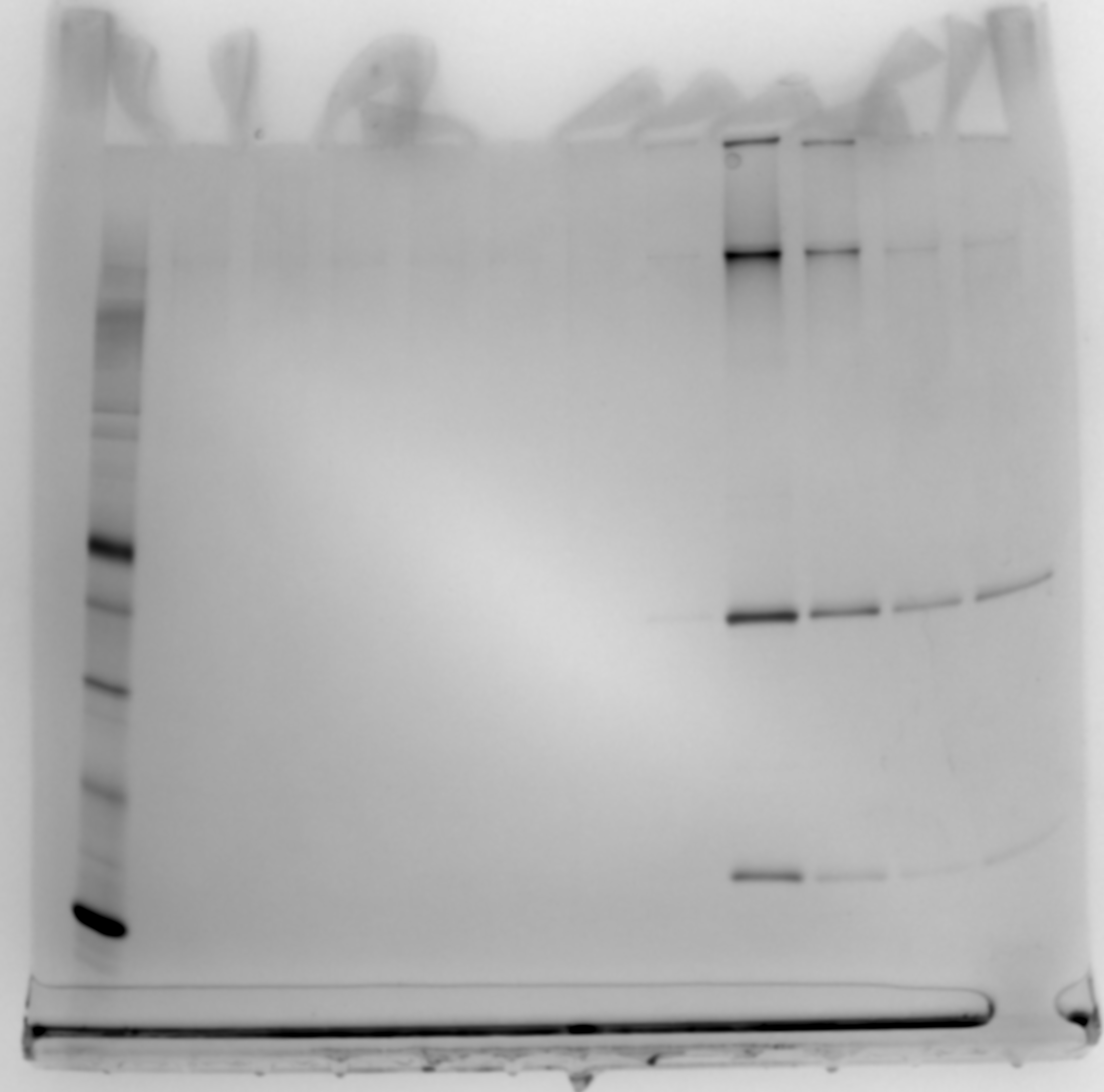

Supplement: Figure 2—figure supplement 1—source data 1. [file elife-64232-fig2-figsupp1-data1.zip › Fig2 S1/Fig2 S1D/090517 DnaC E170Q WB gel 1.tif]

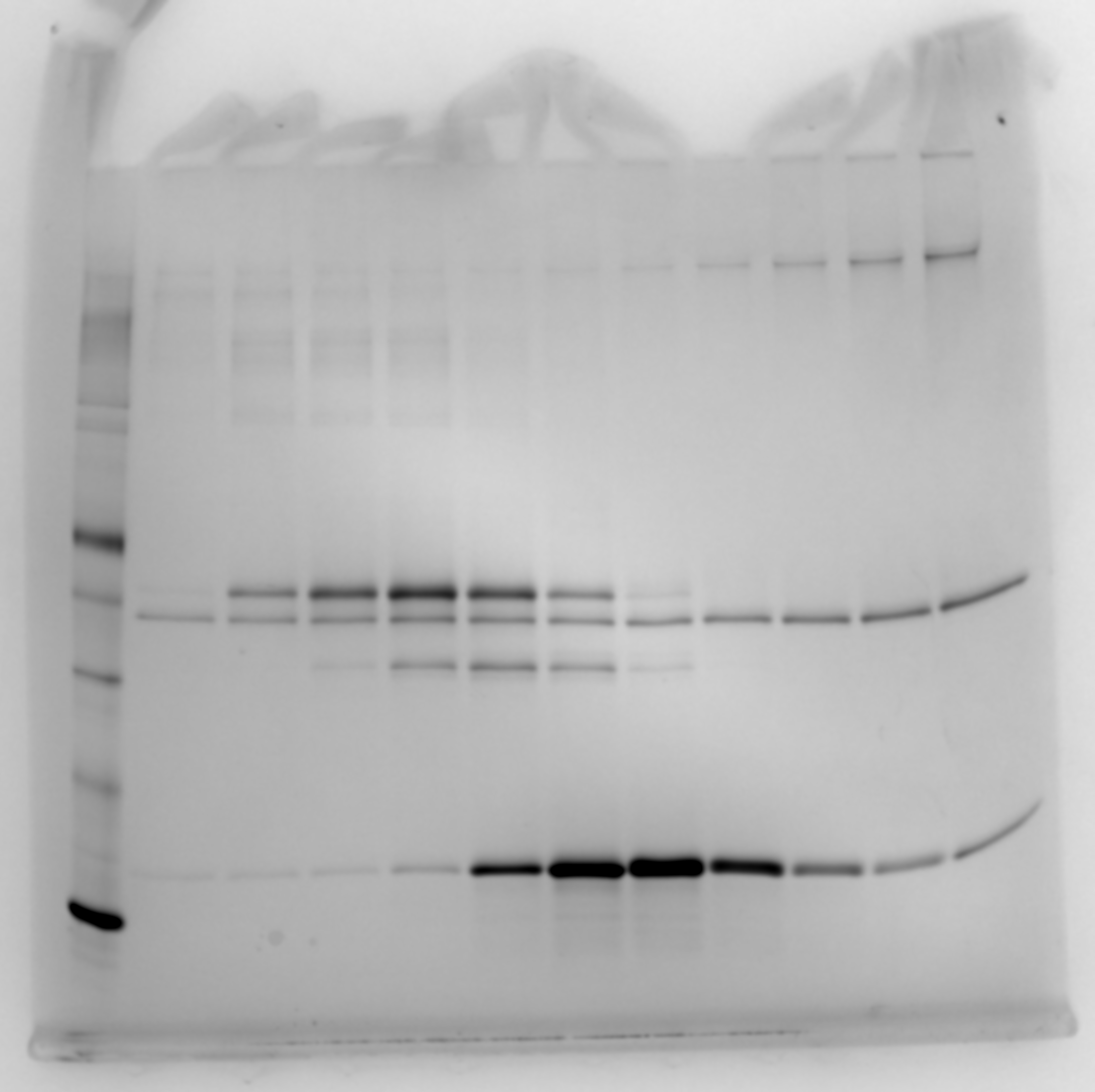

Supplement: Figure 2—figure supplement 1—source data 1. [file elife-64232-fig2-figsupp1-data1.zip › Fig2 S1/Fig2 S1D/090517 DnaC E170Q WB gel 2.tif]

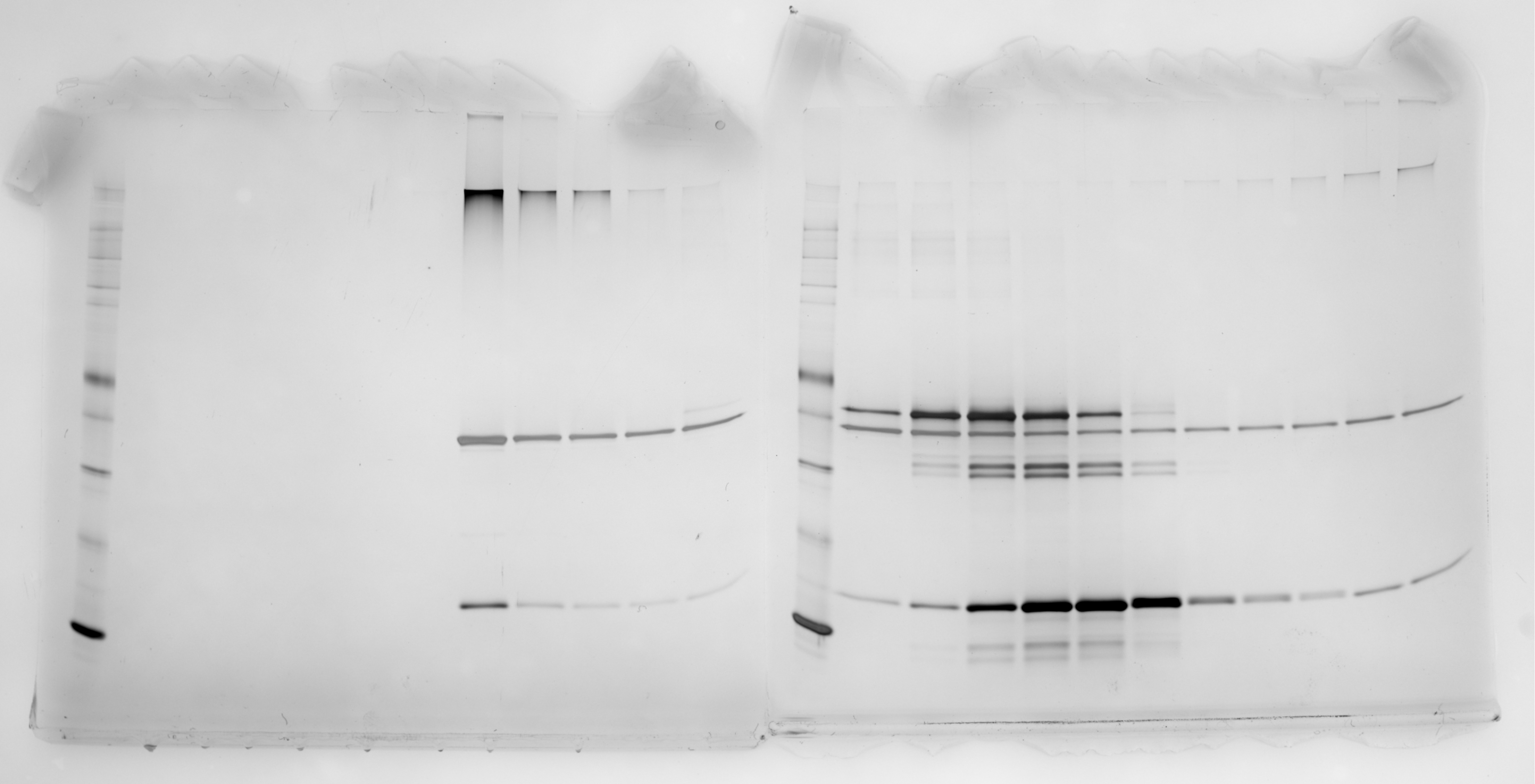

Supplement: Figure 2—figure supplement 1—source data 1. [file elife-64232-fig2-figsupp1-data1.zip › Fig2 S1/Fig2 S1C/072017 N203A new prep.tif]

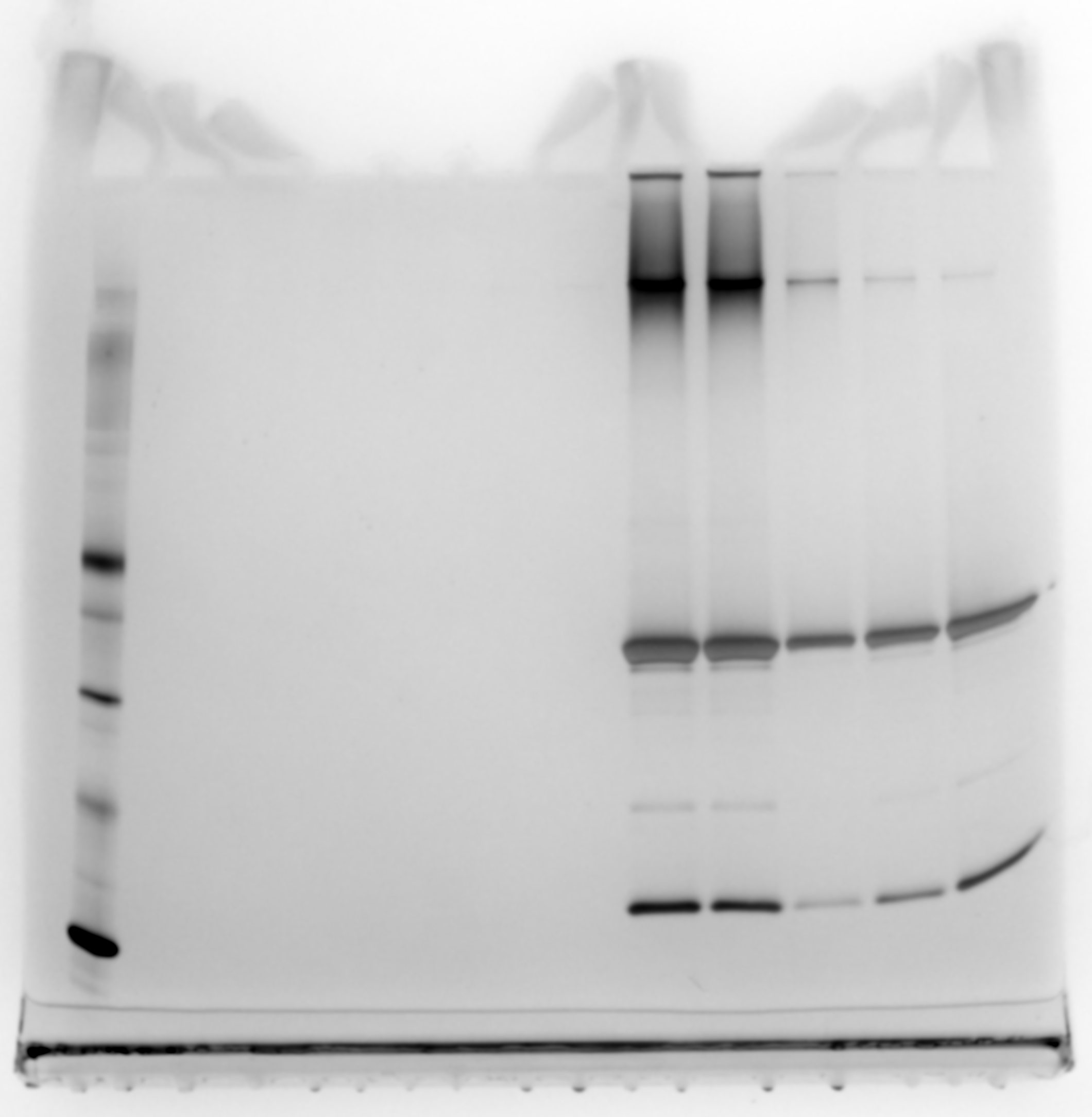

Supplement: Figure 3—source data 1. [file elife-64232-fig3-data1.zip › Fig 3 S1/Fig3 S1A/082217 KAEA B gel 1.tif]

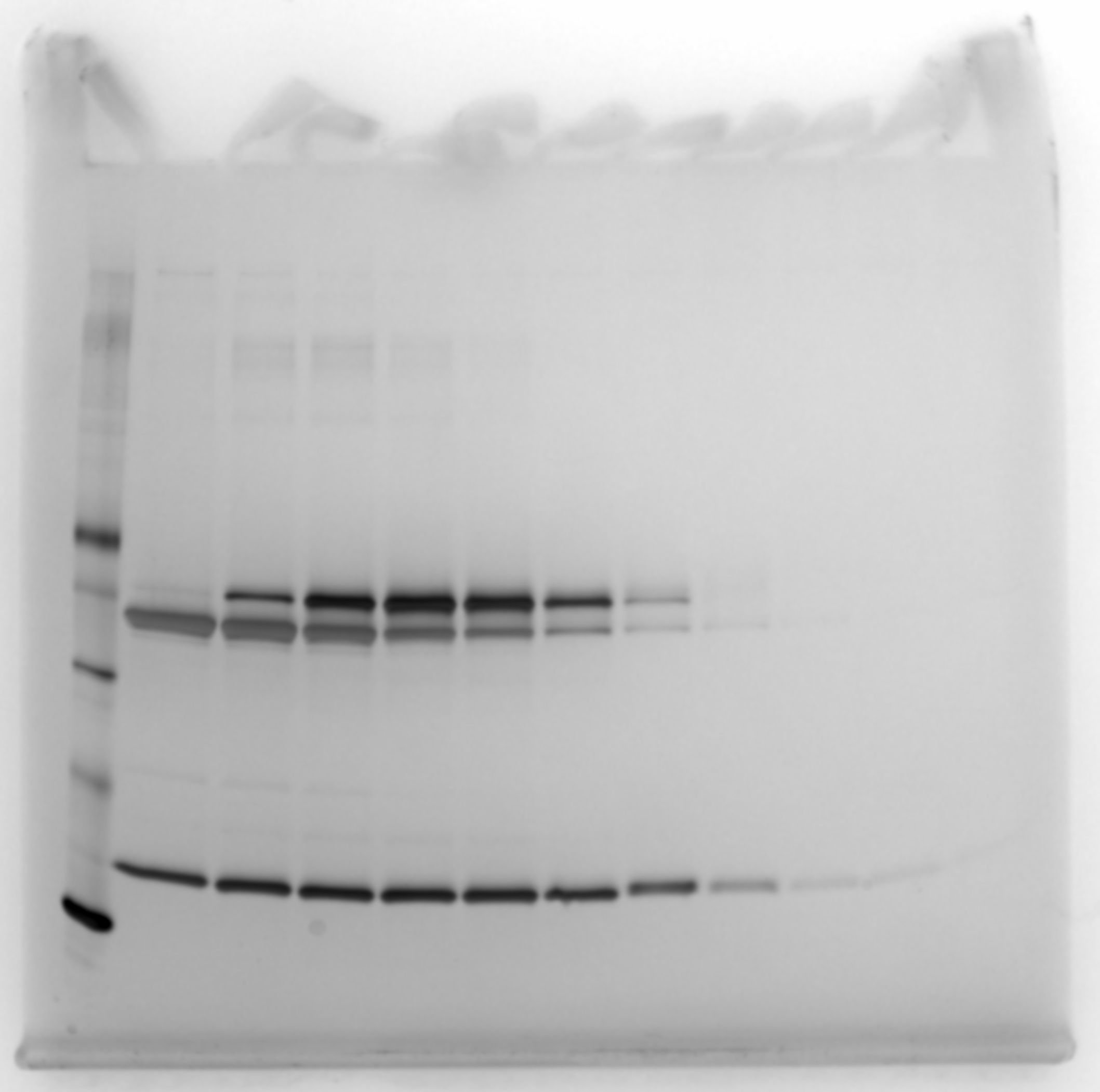

Supplement: Figure 3—source data 1. [file elife-64232-fig3-data1.zip › Fig 3 S1/Fig3 S1A/082217 KAEA B gel 2.tif]

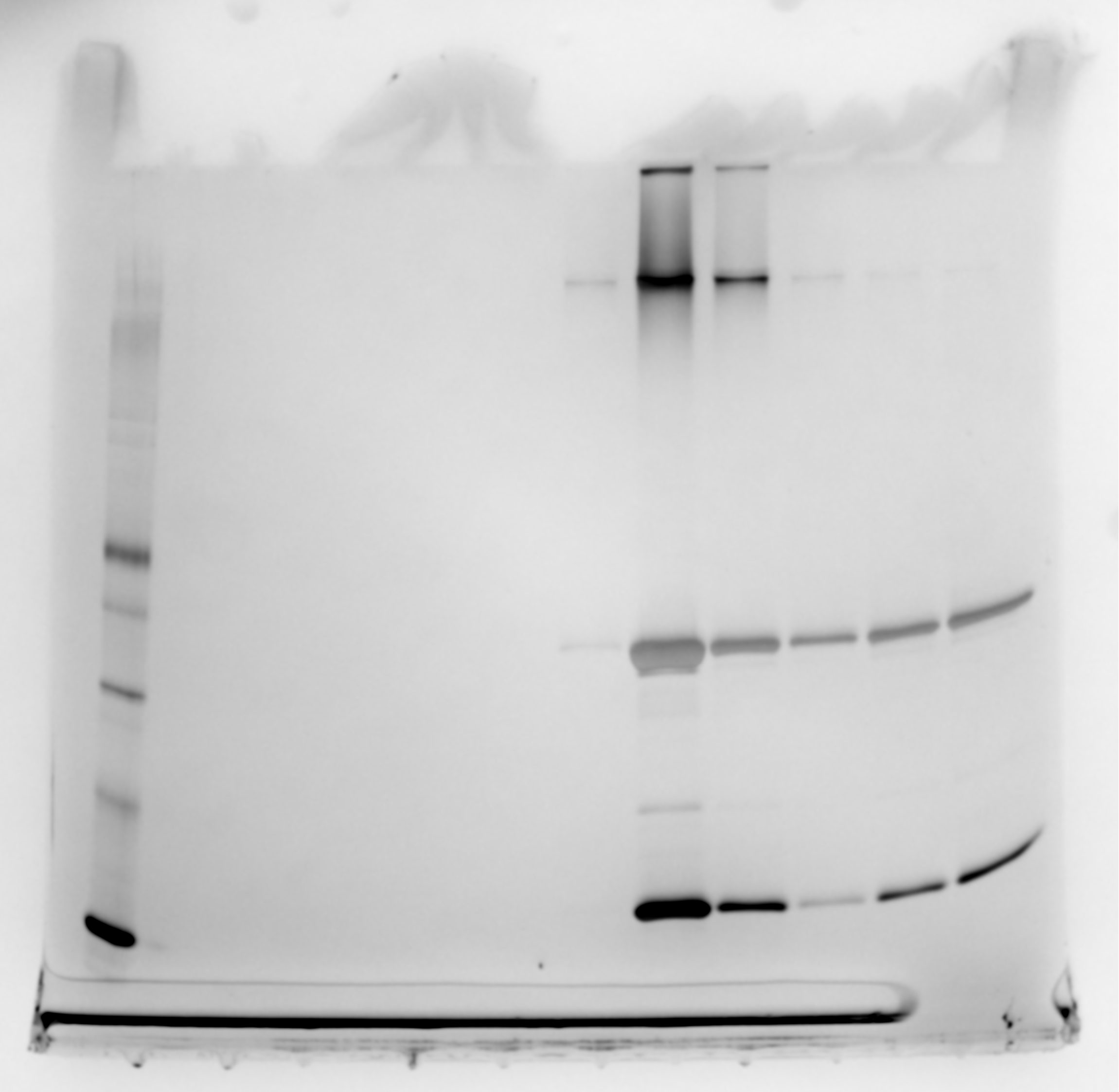

Supplement: Figure 3—source data 1. [file elife-64232-fig3-data1.zip › Fig 3 S1/Fig3 S1E/082417 KAEA B ATP gel 1.tif]

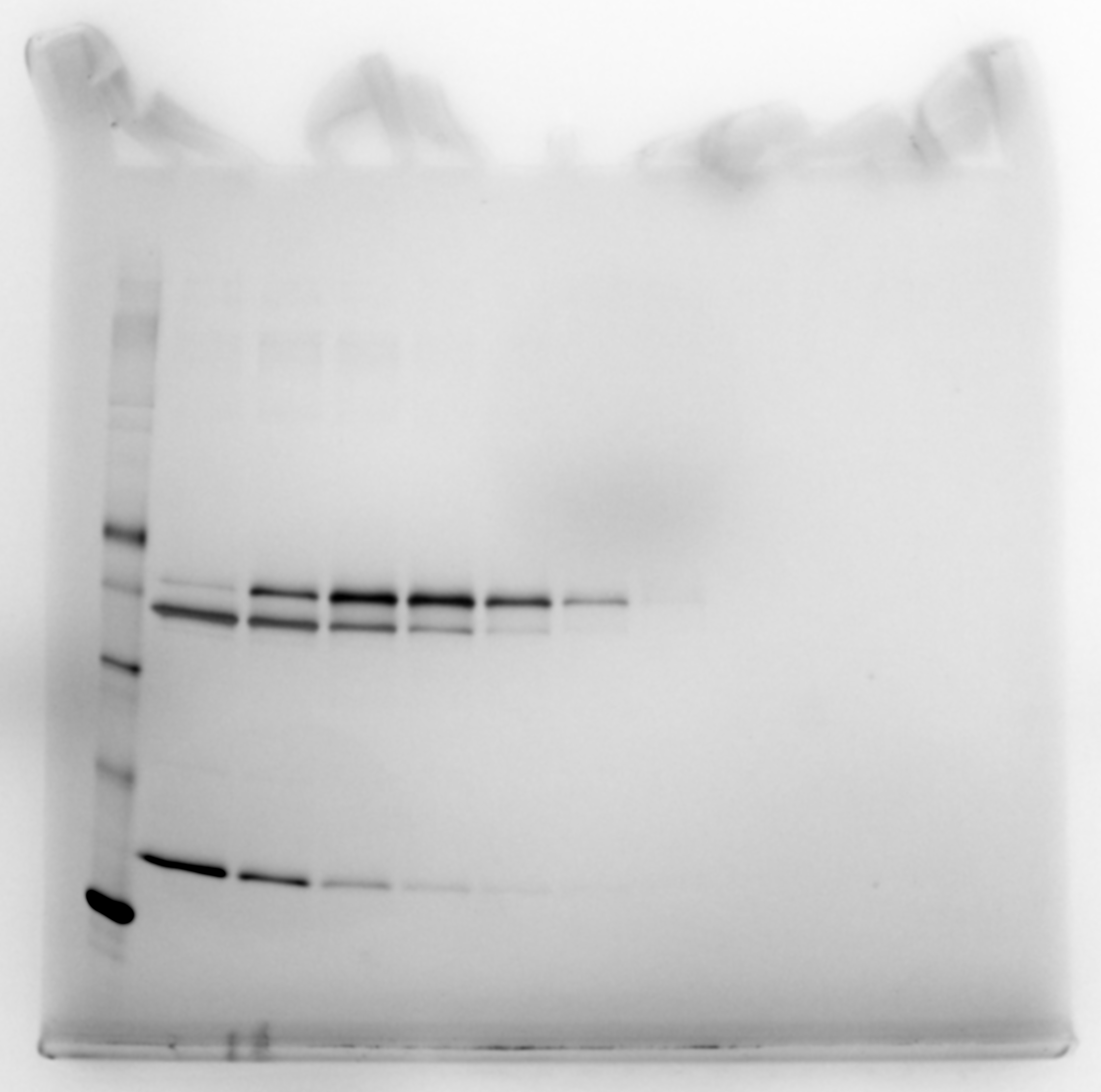

Supplement: Figure 3—source data 1. [file elife-64232-fig3-data1.zip › Fig 3 S1/Fig3 S1E/082417 KAEA B ATP gel 2.tif]

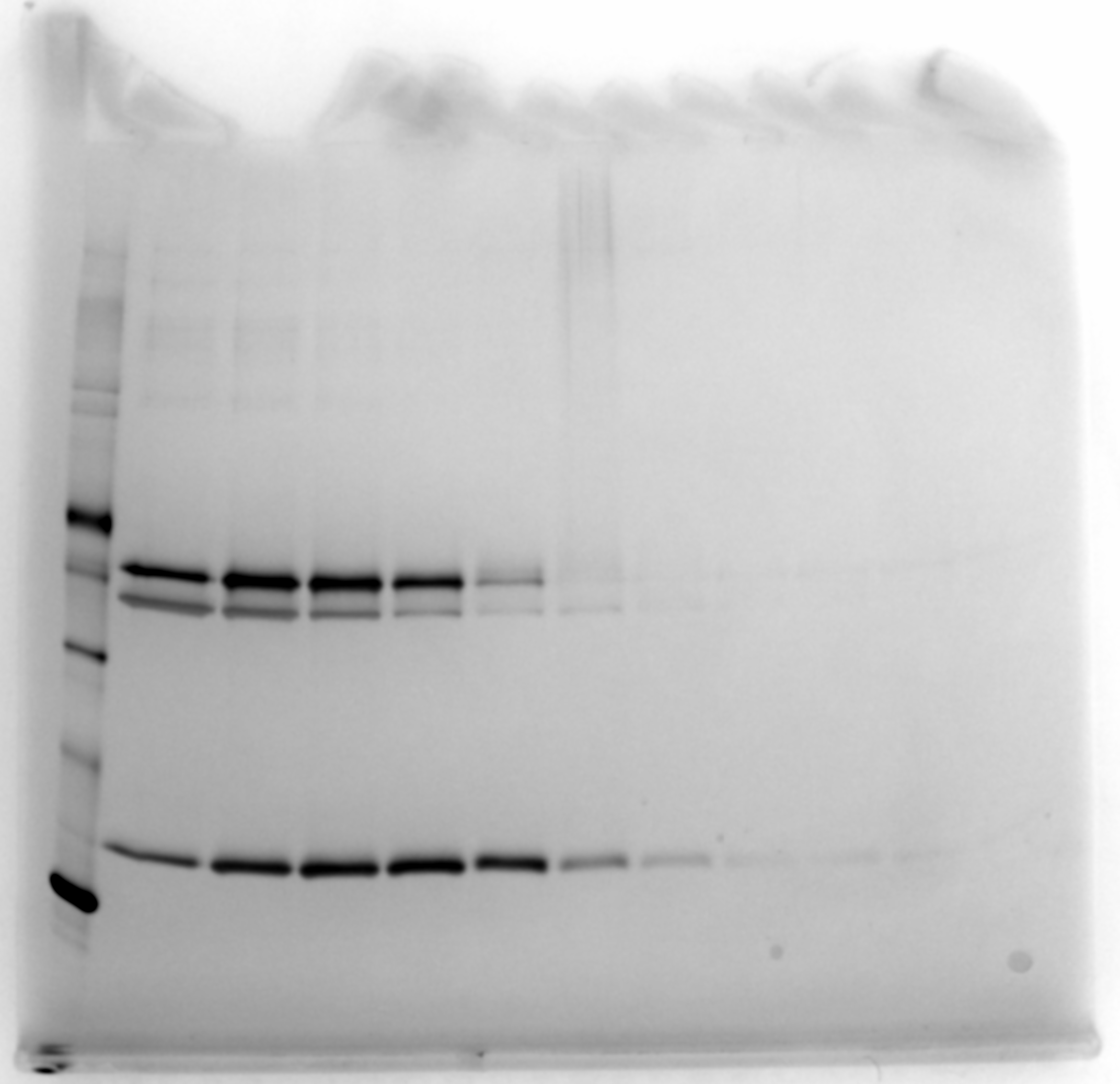

Supplement: Figure 3—source data 1. [file elife-64232-fig3-data1.zip › Fig 3 S1/Fig3 S1B/080417 250 NaCl gel 2.tif]

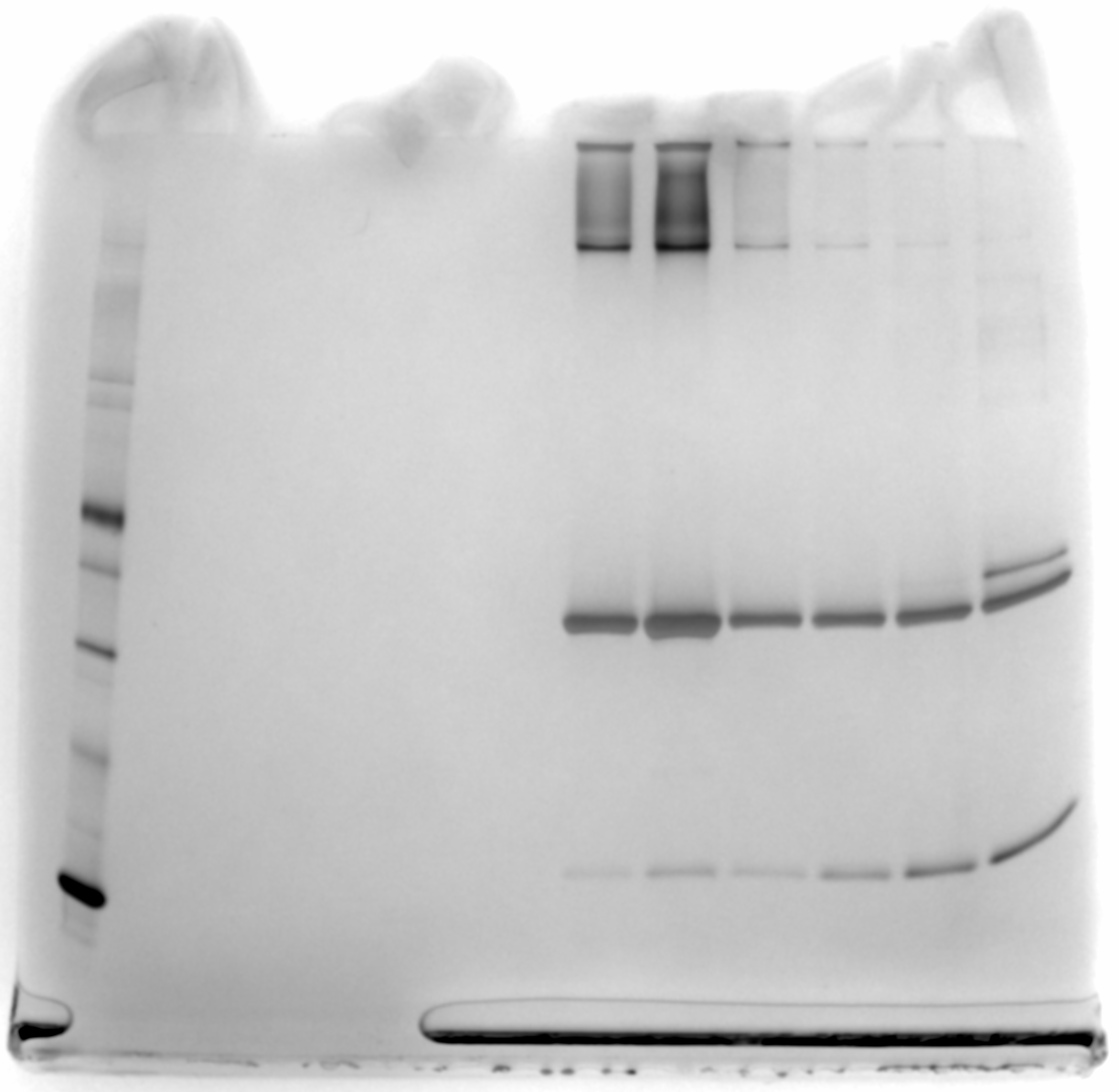

Supplement: Figure 3—source data 1. [file elife-64232-fig3-data1.zip › Fig 3 S1/Fig3 S1B/080417 250 NaCl gel 1.tif]

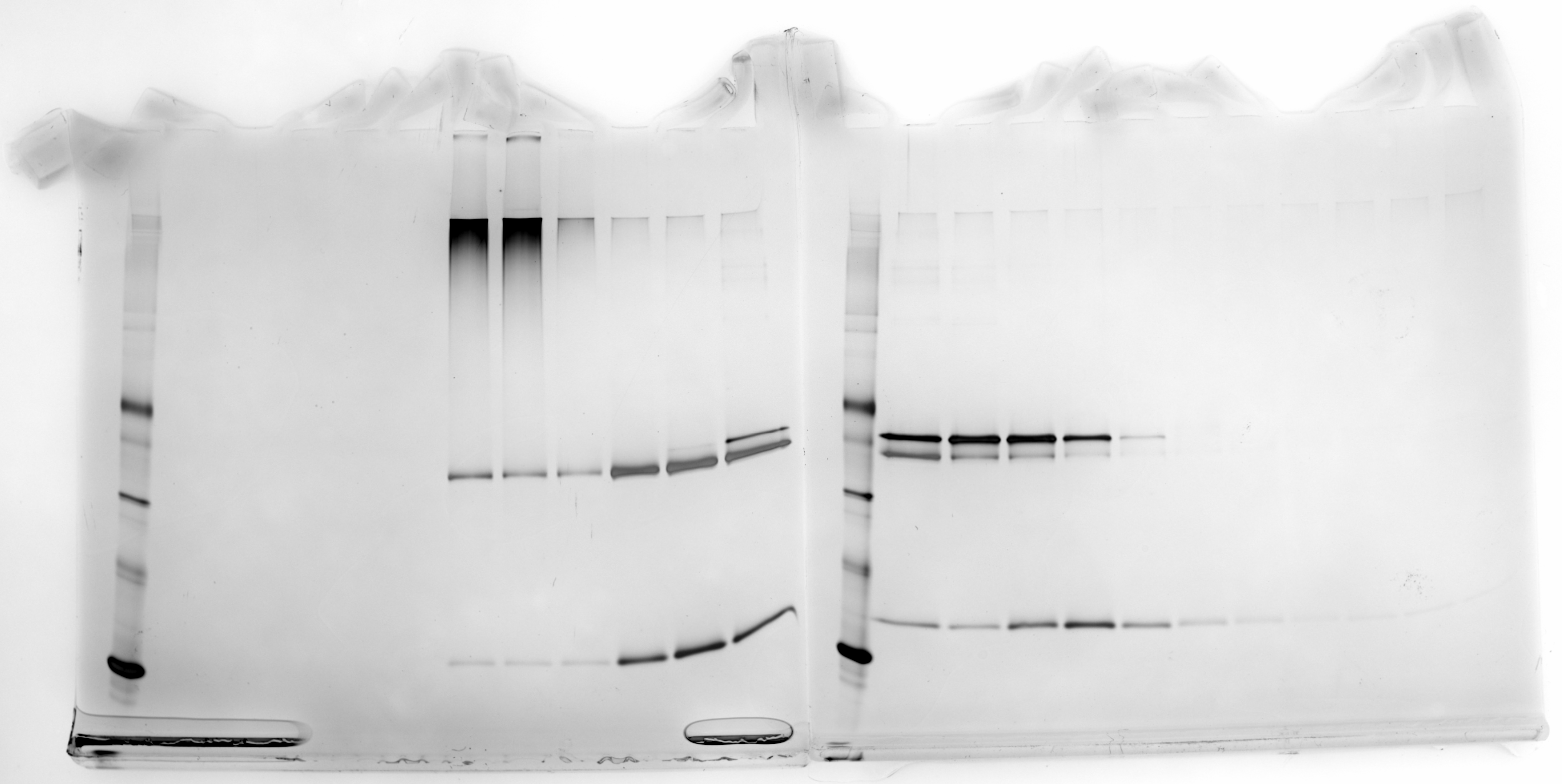

Supplement: Figure 3—source data 1. [file elife-64232-fig3-data1.zip › Fig 3 S1/Fig3 S1C/071917 ATP not ADP.tif]

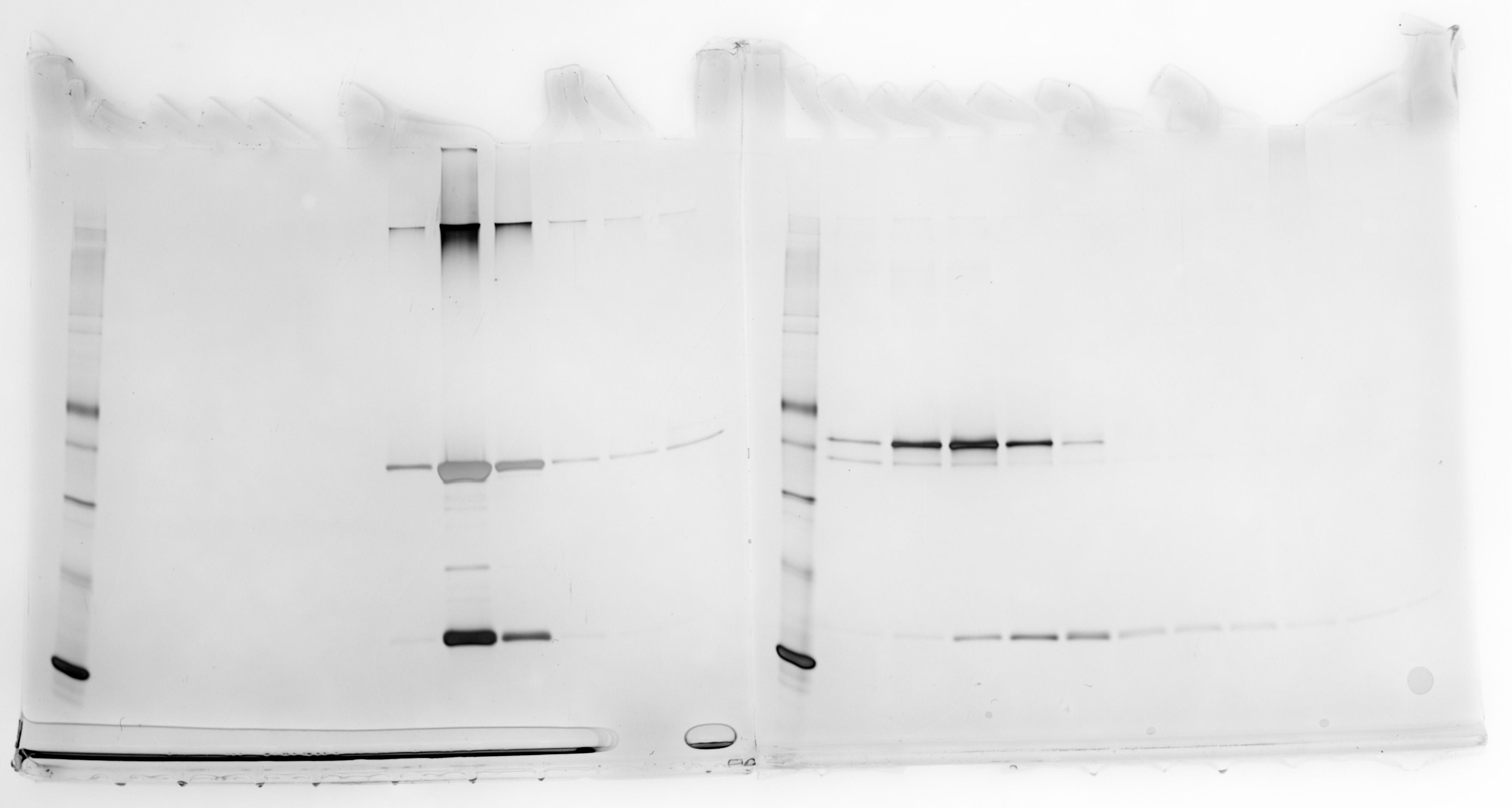

Supplement: Figure 3—source data 1. [file elife-64232-fig3-data1.zip › Fig 3 S1/Fig3 S1D/071917 ADP.tif]

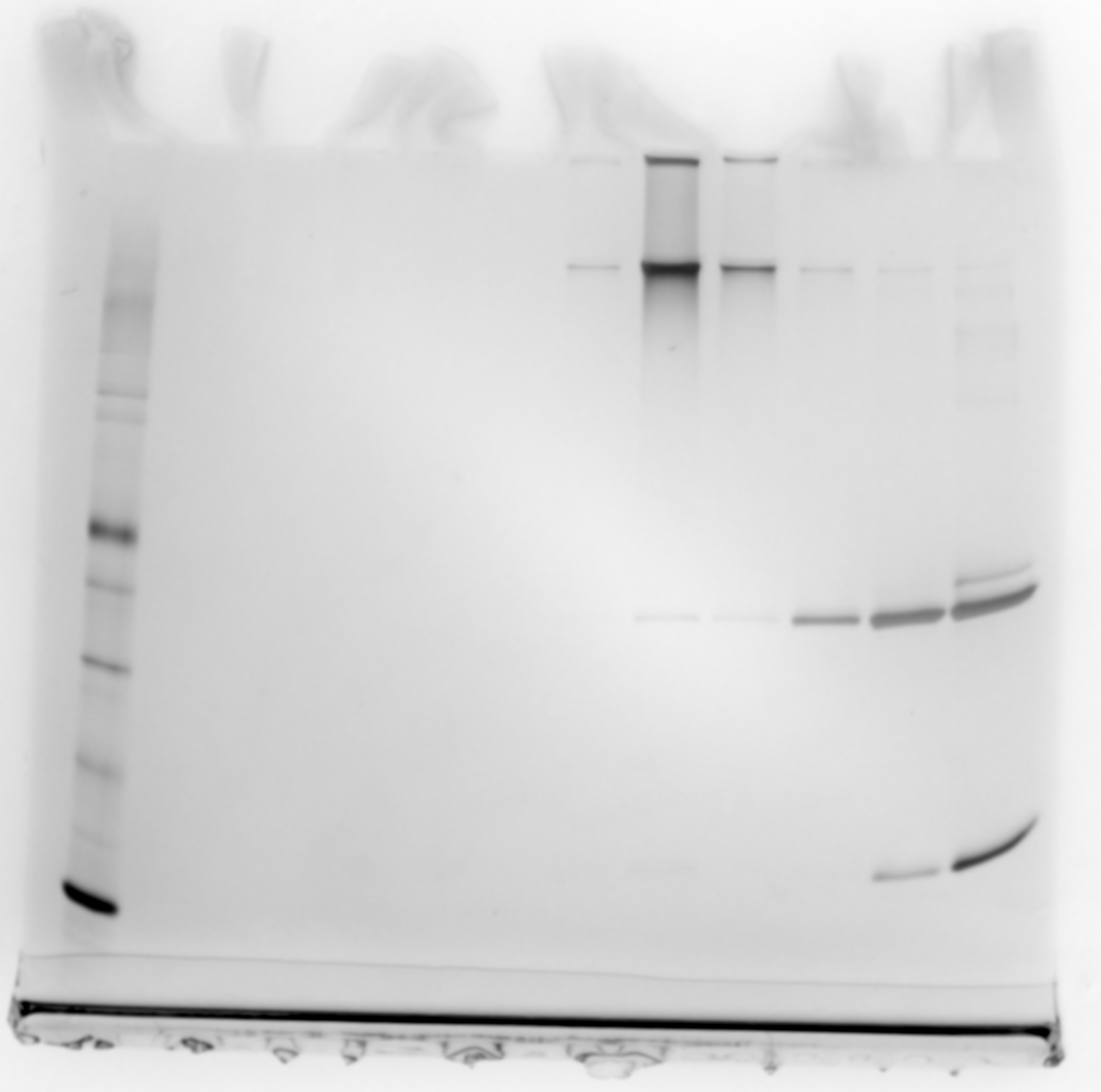

Supplement: Figure 5—figure supplement 1—source data 1. [file elife-64232-fig5-figsupp1-data1.zip › Fig 5 S1/Fig5 S1E/091317 DnaC K143E gel 1.tif]

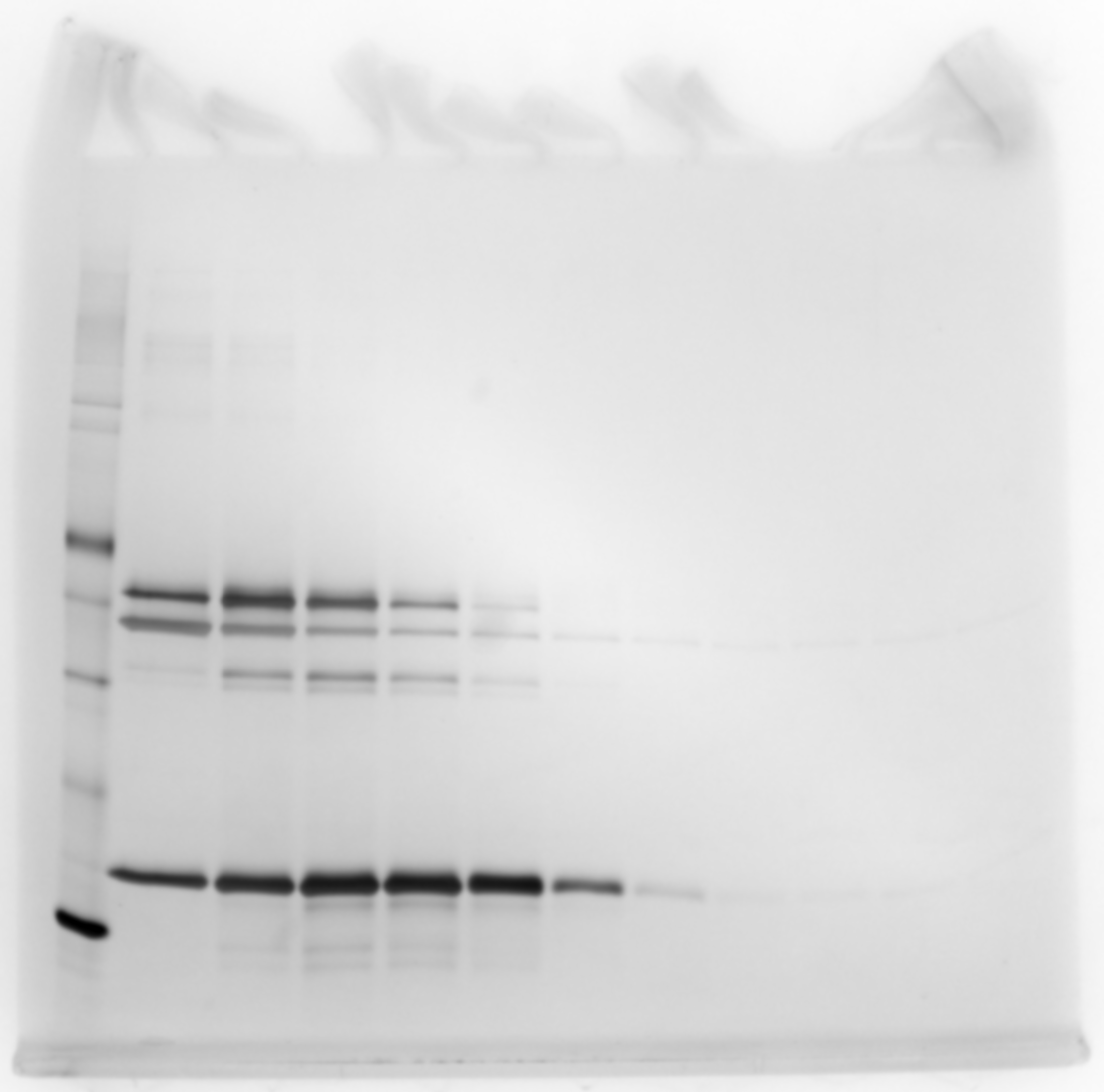

Supplement: Figure 5—figure supplement 1—source data 1. [file elife-64232-fig5-figsupp1-data1.zip › Fig 5 S1/Fig5 S1E/091317 DnaC K143E gel 2.tif]

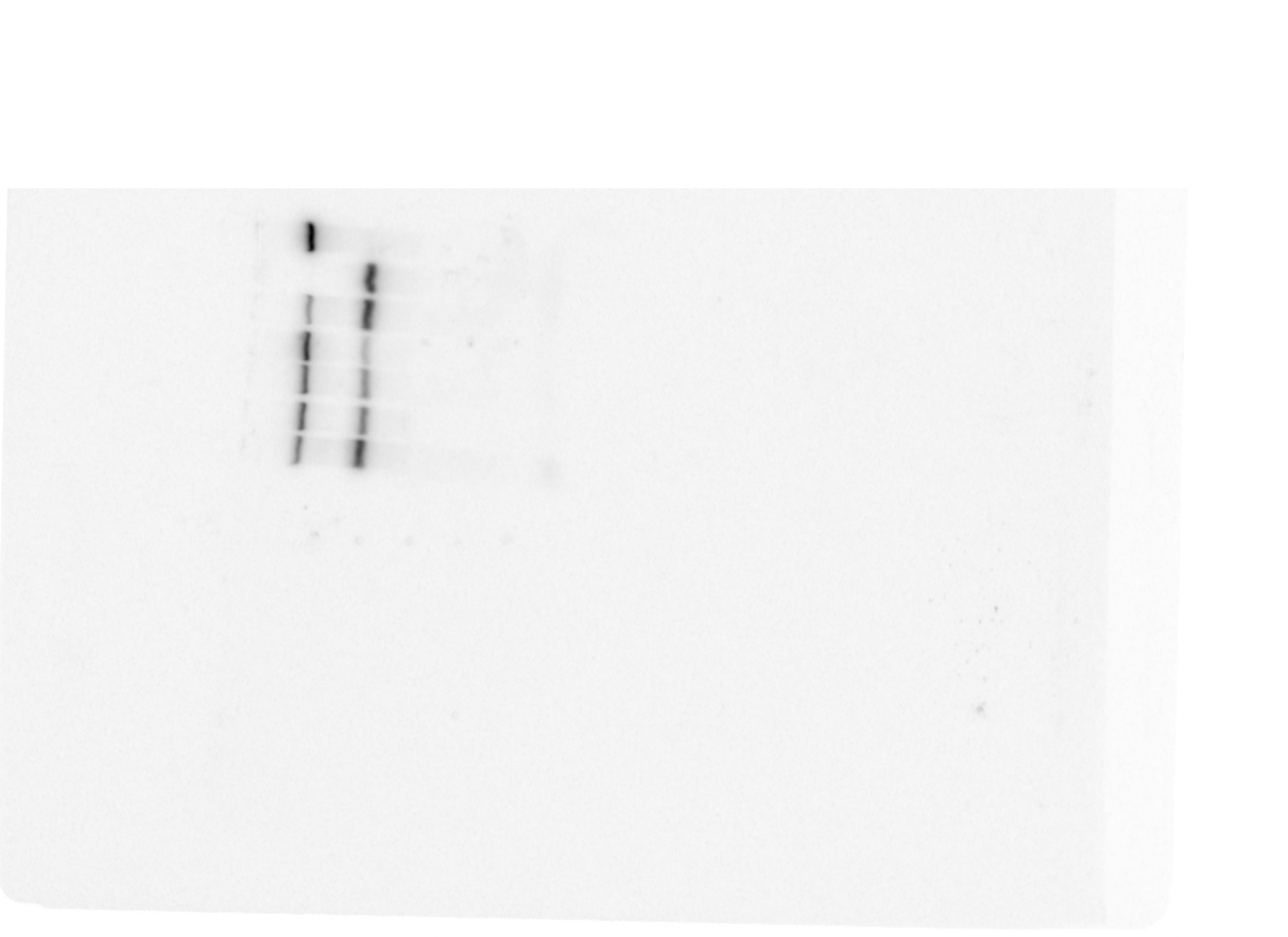

Supplement: Figure 6—figure supplement 1—source data 1. [file elife-64232-fig6-figsupp1-data1.zip › Fig 6 S1/Fig6 S1H.png]

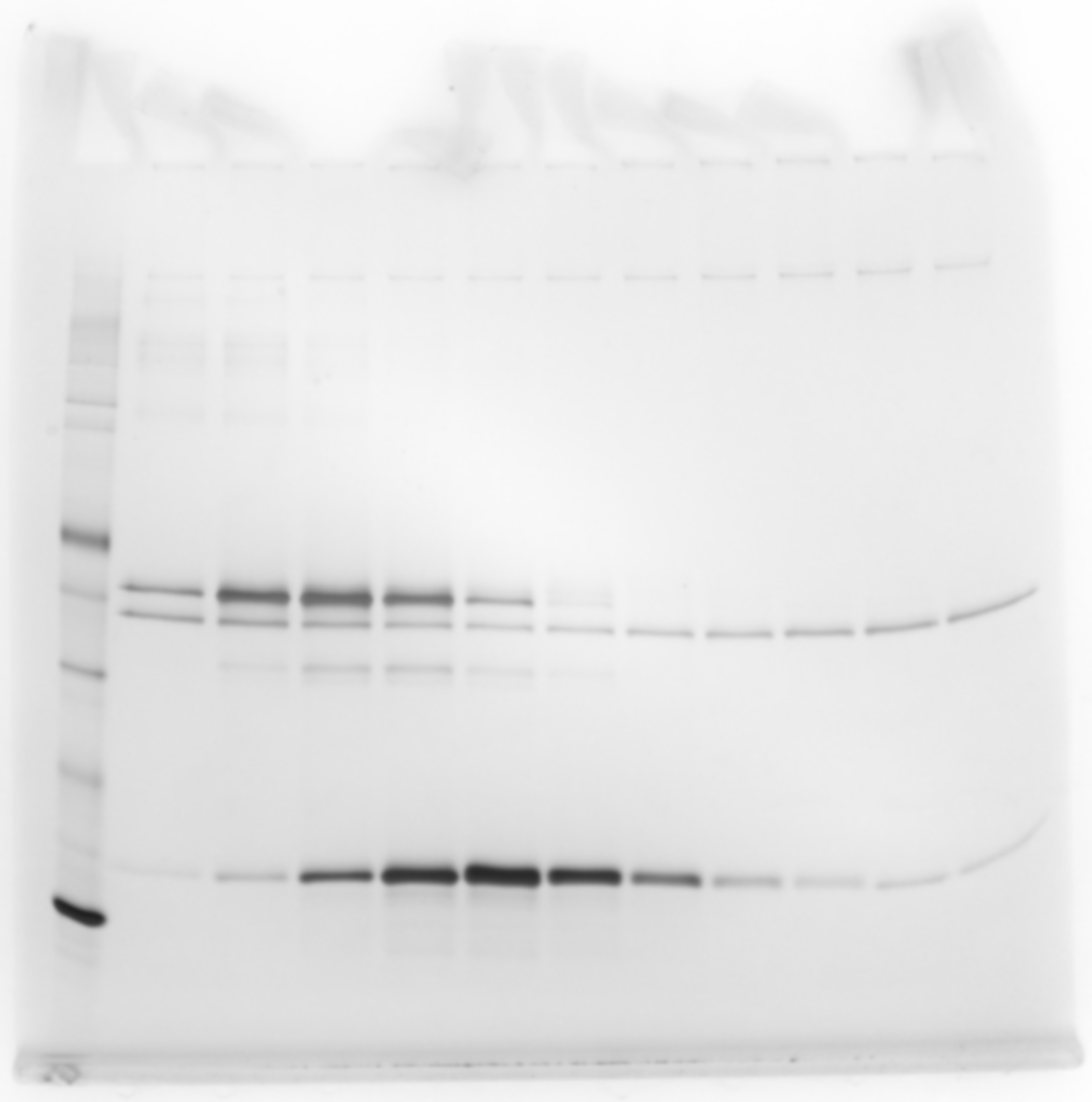

Supplement: Figure 6—figure supplement 1—source data 1. [file elife-64232-fig6-figsupp1-data1.zip › Fig 6 S1/Fig6 S1G/091317 DnaC 810 gel 2.tif]

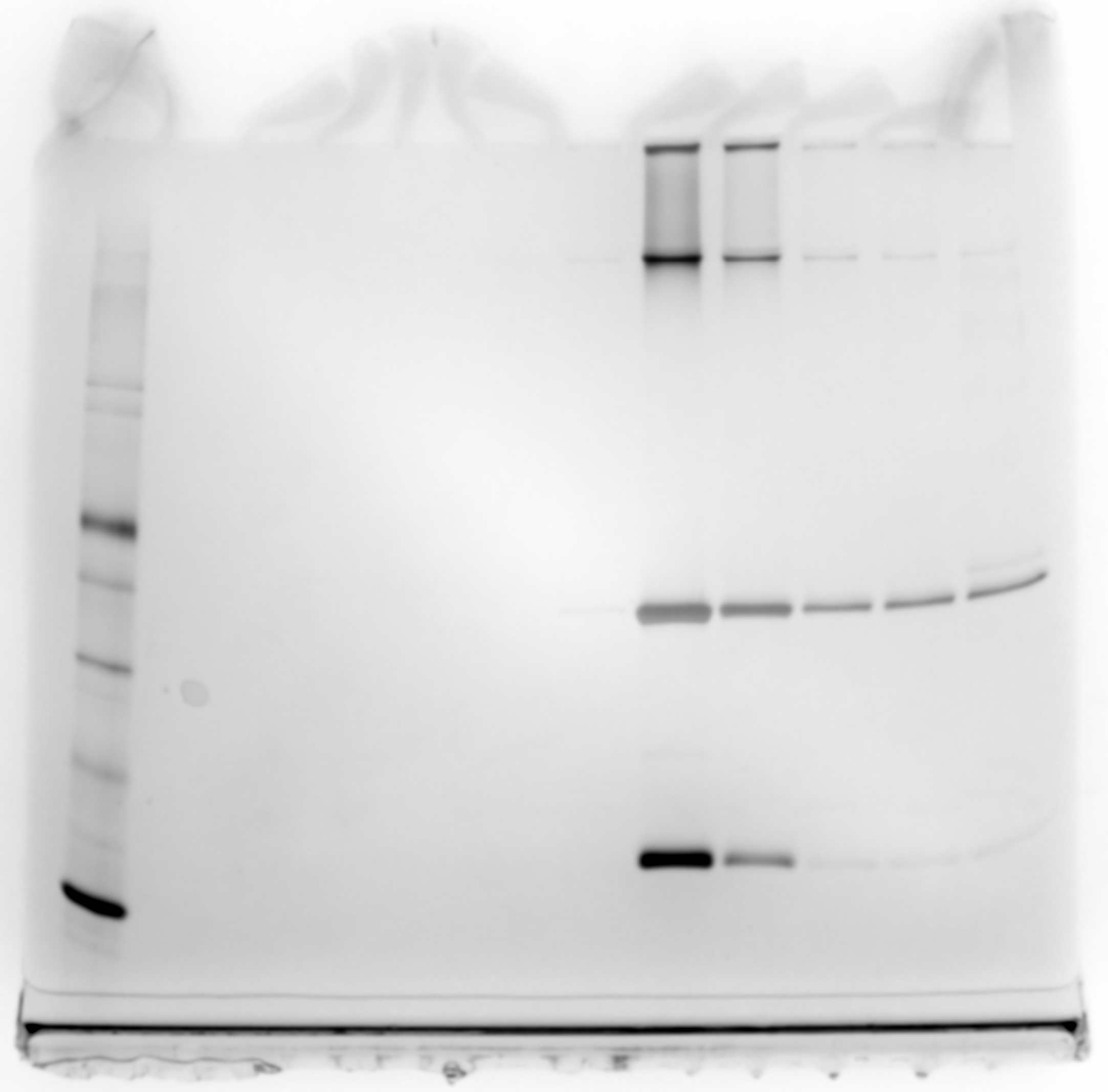

Supplement: Figure 6—figure supplement 1—source data 1. [file elife-64232-fig6-figsupp1-data1.zip › Fig 6 S1/Fig6 S1G/091317 DnaC 810 gel 1.tif]

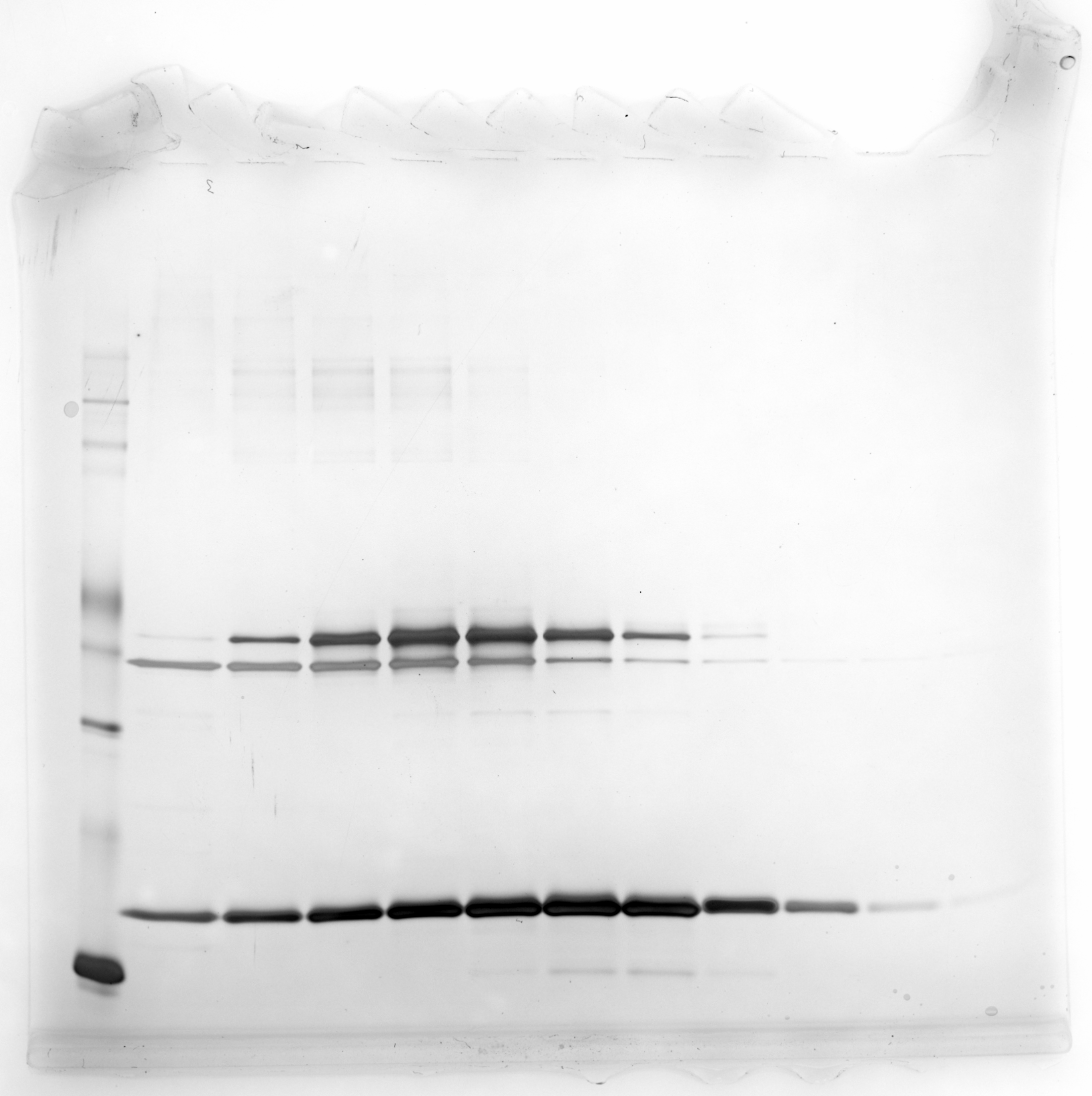

Supplement: Figure 6—figure supplement 1—source data 1. [file elife-64232-fig6-figsupp1-data1.zip › Fig 6 S1/Fig6 S1C/053017 R216A gel2.tif]

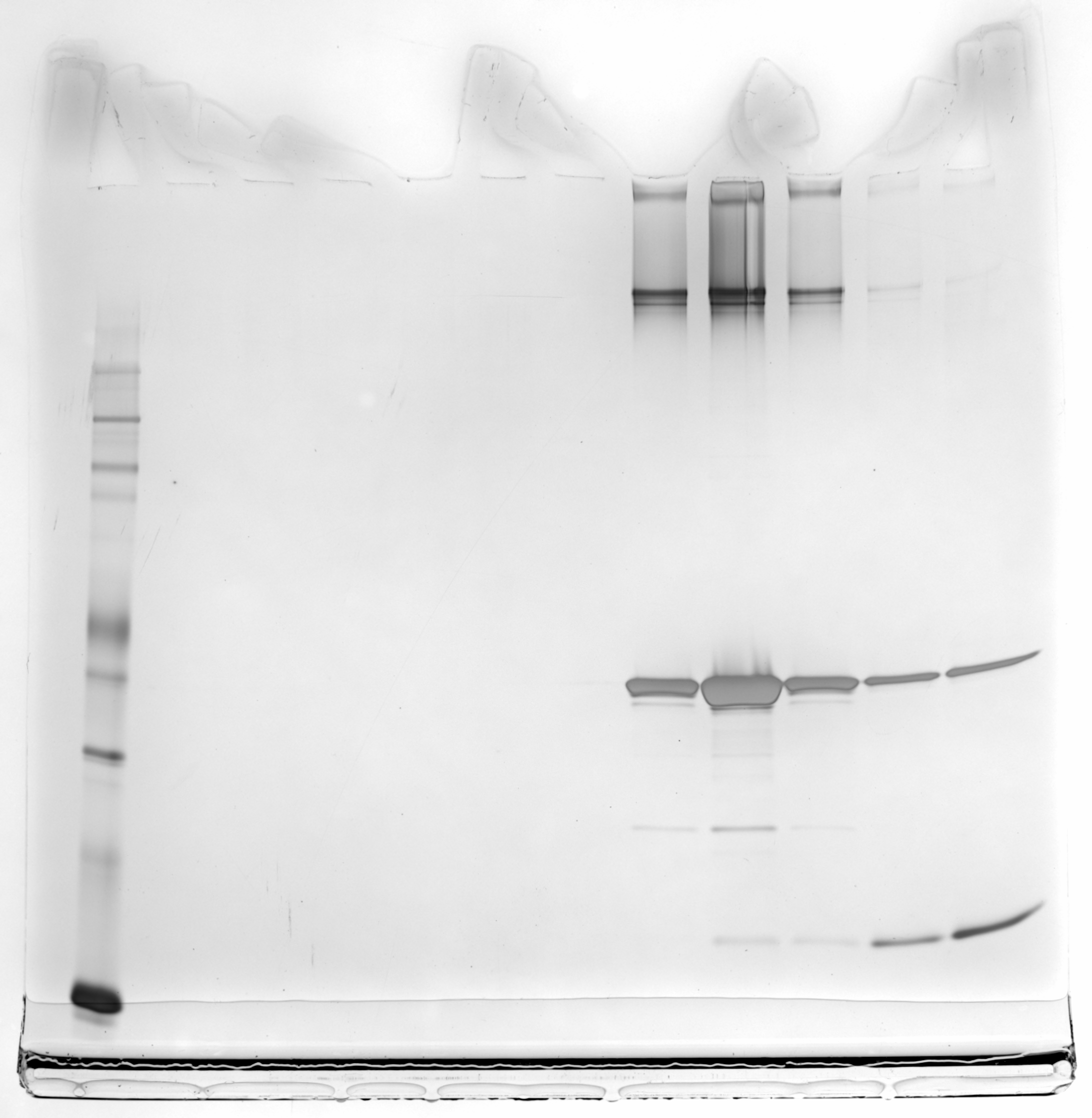

Supplement: Figure 6—figure supplement 1—source data 1. [file elife-64232-fig6-figsupp1-data1.zip › Fig 6 S1/Fig6 S1C/053017 R216A gel1.tif]

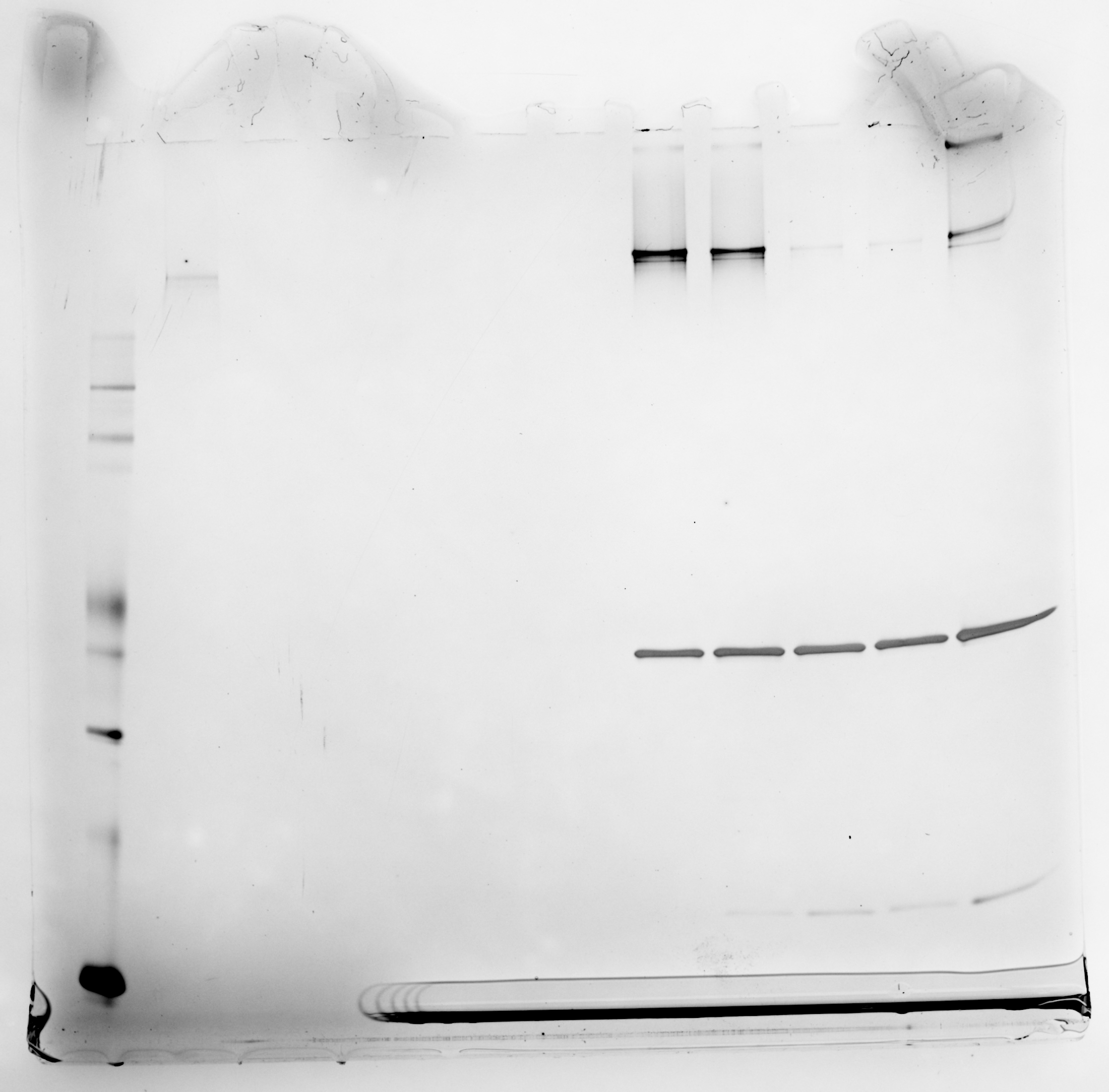

Supplement: Figure 6—figure supplement 1—source data 1. [file elife-64232-fig6-figsupp1-data1.zip › Fig 6 S1/Fig6 S1B/053017 R216D gel1.tif]

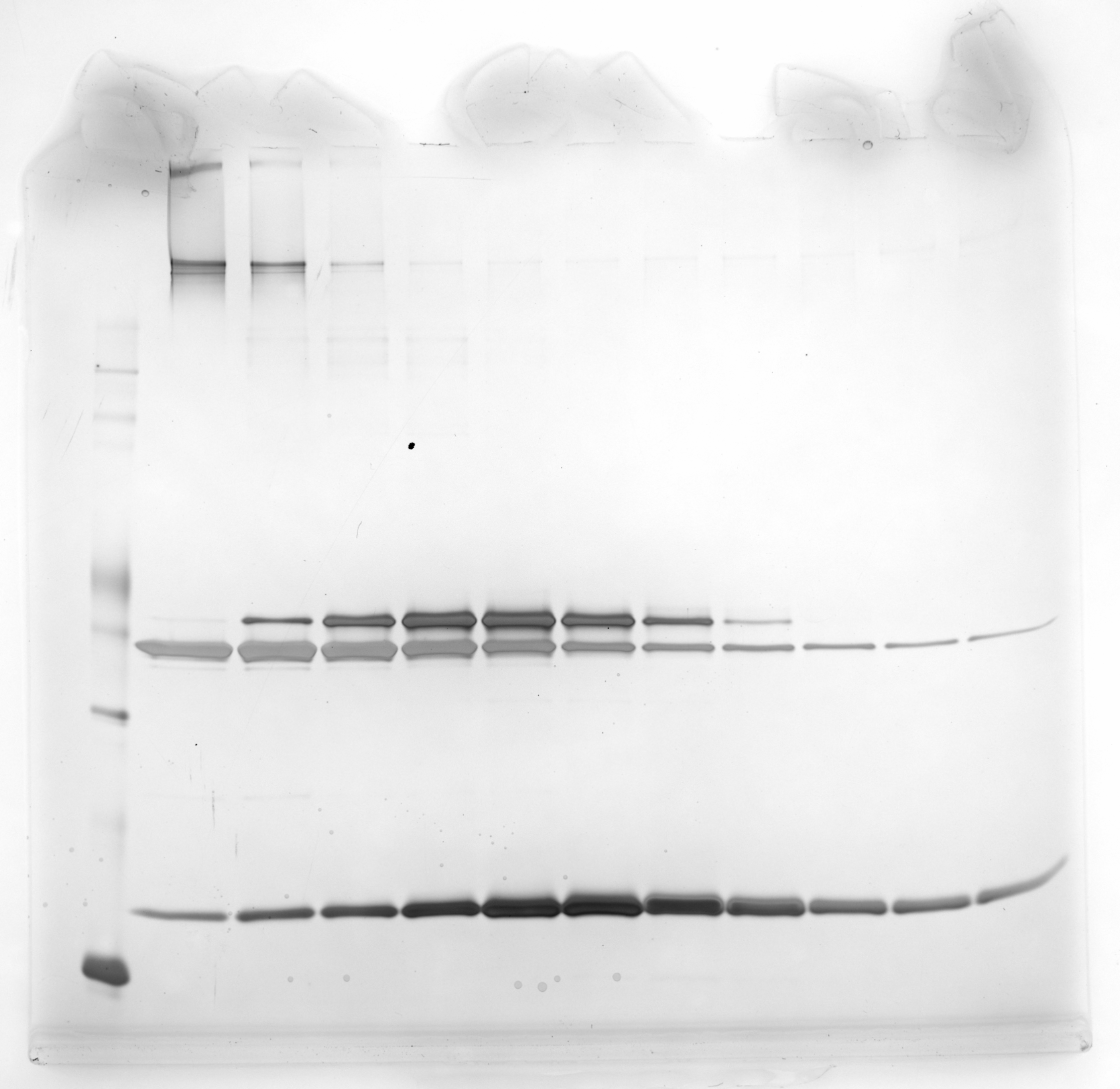

Supplement: Figure 6—figure supplement 1—source data 1. [file elife-64232-fig6-figsupp1-data1.zip › Fig 6 S1/Fig6 S1B/053017 R216D gel2.tif]
